# Influence of Germline Genetics on Tacrolimus Pharmacokinetics and Pharmacodynamics in Allogeneic Hematopoietic Stem Cell Transplant Patients

**DOI:** 10.3390/ijms21030858

**Published:** 2020-01-29

**Authors:** Jing Zhu, Tejendra Patel, Jordan A. Miller, Chad D. Torrice, Mehak Aggarwal, Margaret R. Sketch, Maurice D. Alexander, Paul M. Armistead, James M. Coghill, Tatjana Grgic, Katarzyna J. Jamieson, Jonathan R. Ptachcinski, Marcie L. Riches, Jonathan S. Serody, John L. Schmitz, J. Ryan Shaw, Thomas C. Shea, Oscar Suzuki, Benjamin G. Vincent, William A. Wood, Kamakshi V. Rao, Tim Wiltshire, Eric T. Weimer, Daniel J. Crona

**Affiliations:** 1 The Center for Pharmacogenomics and Individualized Therapy, Division of Pharmacotherapy and Experimental Therapeutics, University of North Carolina Eshelman School of Pharmacy, Chapel Hill, NC 27599, USA; daisy23@email.unc.edu (J.Z.); tejendra.patel@outlook.com (T.P.); chad_torrice@med.unc.edu (C.D.T.); mehaka@email.unc.edu (M.A.); margosketch18@gmail.com (M.R.S.); osuzuki@email.unc.edu (O.S.); timw@unc.edu (T.W.); 2 Department of Pharmacy, University of North Carolina Hospitals and Clinics, Chapel Hill, NC 27599, USA; Jordan.Miller@unchealth.unc.edu (J.A.M.); Maurice.Alexander@unchealth.unc.edu (M.D.A.); Tatjana.Grgic@unchealth.unc.edu (T.G.); Jonathan.Ptachcinski@unchealth.unc.edu (J.R.P.); Ryan.Shaw@unchealth.unc.edu (J.R.S.); Kamakshi.Rao@unchealth.unc.edu (K.V.R.); 3 Division of Practice Advancement and Clinical Education, University of North Carolina Eshelman School of Pharmacy, Chapel Hill, NC 27599, USA; 4 Division of Hematology and Oncology, Department of Medicine, University of North Carolina, Chapel Hill, NC 27599, USA; paul_armistead@med.unc.edu (P.M.A.); jcoghill@email.unc.edu (J.M.C.); katarzyna_jamieson@med.unc.edu (K.J.J.); marcie_riches@med.unc.edu (M.L.R.); jonathan_serody@med.unc.edu (J.S.S.); tom_shea@med.unc.edu (T.C.S.); benjamin_vincent@med.unc.edu (B.G.V.); william_wood@med.unc.edu (W.A.W.); 5 Lineberger Comprehensive Cancer Center, University of North Carolina, Chapel Hill, NC 27599, USA; 6 Department of Pathology & Laboratory Medicine, University of North Carolina at Chapel Hill School of Medicine, Chapel Hill, NC 27599, USA; John.Schmitz@unchealth.unc.edu (J.L.S.); Eric.Weimer@unchealth.unc.edu (E.T.W.)

**Keywords:** tacrolimus, pharmacogenetics, pharmacokinetics, pharmacodynamics, allogeneic hematopoietic stem cell transplant, single nucleotide polymorphism (SNP), germline, CYP3A4/5, ABCB1

## Abstract

Tacrolimus exhibits high inter-patient pharmacokinetics (PK) variability, as well as a narrow therapeutic index, and therefore requires therapeutic drug monitoring. Germline mutations in cytochrome P450 isoforms 4 and 5 genes (*CYP3A4/5*) and the ATP-binding cassette B1 gene (*ABCB1*) may contribute to interindividual tacrolimus PK variability, which may impact clinical outcomes among allogeneic hematopoietic stem cell transplantation (HSCT) patients. In this study, 252 adult patients who received tacrolimus for acute graft versus host disease (aGVHD) prophylaxis after allogeneic HSCT were genotyped to evaluate if germline genetic variants associated with tacrolimus PK and pharmacodynamic (PD) variability. Significant associations were detected between germline variants in *CYP3A4/5* and *ABCB1* and PK endpoints (e.g., median steady-state tacrolimus concentrations and time to goal tacrolimus concentration). However, significant associations were not observed between *CYP3A4/5* or *ABCB1* germline variants and PD endpoints (e.g., aGVHD and treatment-emergent nephrotoxicity). Decreased age and CYP3A5*1/*1 genotype were independently associated with subtherapeutic tacrolimus trough concentrations while CYP3A5*1*3 or CYP3A5*3/*3 genotypes, myeloablative allogeneic HSCT conditioning regimen (MAC) and increased weight were independently associated with supratherapeutic tacrolimus trough concentrations. Future lines of prospective research inquiry are warranted to use both germline genetic and clinical data to develop precision dosing tools that will optimize both tacrolimus dosing and clinical outcomes among adult HSCT patients.

## 1. Introduction

Allogeneic hematopoietic stem cell transplantation (HSCT) is a widely available and curative treatment option for patients with both malignant and non-malignant hematologic diseases [1,2]. However, acute graft versus host disease (aGVHD) is one of the major life-threatening sequelae associated with allogeneic HSCT. aGVHD occurs when donor transplanted immunocompetent T cells (i.e., “the graft”) identify cells from the HSCT recipient (i.e., “the host”) as foreign, and initiate an immune reaction in the host [3]. Moreover, it has been reported that early onset of aGVHD is associated with increased infection-related mortality [4]. Therefore, optimal pharmacologic immunosuppression is essential to reduce aGVHD risk, and its associated risk of morbidity and mortality [5].

Tacrolimus, a calcineurin inhibitor, is the cornerstone of a multi-drug immunosuppression treatment strategy used to reduce aGVHD risk [6]. Calcineurin inhibition leads to decreased *IL-2* gene transcription and inhibits T cell activation [7]. Moreover, a higher incidence of treatment-emergent nephrotoxicity has been associated with supratherapeutic tacrolimus levels within the first 2 weeks post-transplant [8,9,10,11]. Tacrolimus is characterized by a narrow therapeutic index, and substantial interindividual pharmacokinetic (PK) variability with standard-of-care weight-based dosing [12,13,14]. Therefore, therapeutic drug monitoring (TDM) is required to ensure patients achieve therapeutic trough concentrations within a target range (e.g., 5–15 ng/mL). However, achieving and maintaining target tacrolimus trough concentrations can often be problematic, despite reactive adjustments to frequent tacrolimus TDM.

Tacrolimus has a highly variable absorption profile following oral administration, with an average oral bioavailability of 25%, ranging from 5% to 93% [12,15,16]. Tacrolimus is subjected to extensive hepatic metabolism, where <1% of the parent drug is excreted unchanged [12,17]. Cytochrome P450 isoforms 3A4 and 3A5 (CYP3A4/5) are the primary phase 1 metabolic enzymes responsible for tacrolimus hepatic clearance [18]. Tacrolimus is a substrate for P-glycoprotein (P-gp), which is an important membrane efflux pump that transports drugs out of cells [19], and contributes to a substantial portion of tacrolimus PK variability [20]. Interindividual tacrolimus PK variability can be at least partially explained by clinical and demographic factors, including age, race, hepatic and renal function, and concomitant medications [21].

Interindividual tacrolimus PK variability has also been associated with germline genetic variants among transplant patients [22,23,24]. Recently, there has been considerable interest in the identification and validation of germline genetic variants in *CYP3A4/5* to personalize tacrolimus dosing and improve clinical outcomes. It has been estimated that single nucleotide polymorphisms (SNPs) in *CYP3A5* could explain up to 40% to 50% of the interindividual tacrolimus PK variability [25,26]. In addition, CYP3A4 is the most abundant cytochrome P450 enzyme in human hepatocytes and is also responsible for tacrolimus metabolism. Two intragenic *CYP3A4* SNPs have been hypothesized to contribute to tacrolimus interindividual PK variability [27,28]. In addition to SNPs in genes that encode proteins that influence tacrolimus metabolism, germline variants in drug transporters may also contribute to tacrolimus PK variability. *ABCB1* encodes P-gp, and it is highly expressed in both the enterocytes and hepatocytes, and thus *ABCB1* SNPs could explain interindividual tacrolimus absorption and exposure [29]. However, P-gp is also located on the apical membrane of renal tubular epithelial cells, and SNPs have been associated with increased risk of tacrolimus-induced nephrotoxicity [30,31,32].

The most recent guidelines from The Clinical Pharmacogenetics Implementation Consortium (CPIC) have a rich set of recommendations for pharmacogenetically guided tacrolimus dosing [24]. These recommendations come from experience in solid organ transplant patients, and there is a lack of evidence in the allogeneic HSCT patient population to apply the CPIC recommendations. While recent publications have begun to address CYP3A4/5-guided tacrolimus dosing in allogeneic HSCT, these pharmacogenetic studies have either focused on intravenous administration of tacrolimus [30], or underrepresented black patients in the studies [11,33,34]. Importantly, the CYP3A5*3 variant minor allele frequency (MAF) varies across races, and is estimated to be as high as 95% among white patients but only approximately 33% in black patients [35]. Therefore, there is still an unmet clinical and public health need to optimize tacrolimus dosing, particularly among black patients. To address this unmet need, this pharmacogenetics study sought to evaluate associations between *CYP3A4/5* and *ABCB1* SNPs and PK/pharmacodynamic (PD) endpoints, which include median steady-state tacrolimus concentration, time to therapeutic tacrolimus concentration, incidence and severity of aGVHD, and treatment-emergent nephrotoxicity.

## 2. Results

**Figure 1:**
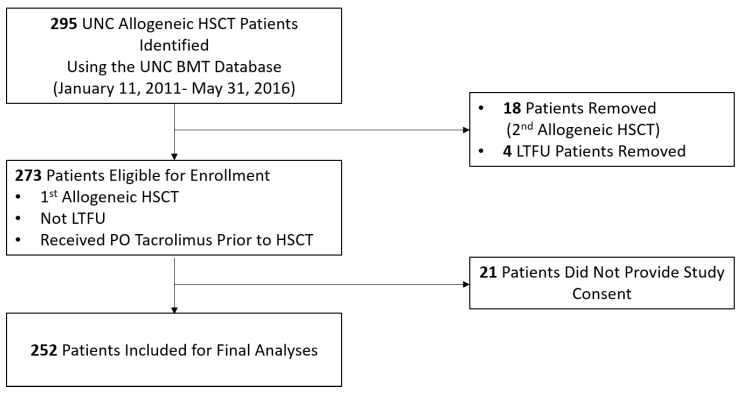
Study schematic. Genomic DNA from patients at our institution who received allogeneic hematopoietic stem cell transplant between January 11, 2011 and May 31, 2016 (*n* = 252) was used to test associations between three single nucleotide polymorphisms (SNPs) in *CYP3A4/5* and three SNPs in *ABCB1* with pharmacokinetics and pharmacodynamics endpoints. Abbreviations: aGVHD, acute graft versus host disease; BMT, bone marrow transplant; HSCT, hematopoietic stem cell transplant; LTFU, lost to follow-up; PO, oral; UNC, University of North Carolina.

A total of 295 adult allogeneic HSCT patients were identified by the University of North Carolina (UNC) Bone Marrow Transplant (BMT) Program database, and a total of 252 were enrolled and included in the final analyses (Table 1, Figure 1). Median age at the time of allogeneic HSCT was 52 years (range 19–76), 58% of the patients were male, and 84% were white. The majority of patients received a transplant that used peripheral blood stem cells (PBSCs) as the stem cell source (94%), from matched unrelated donors (MUDs) (65%), and received a myeloablative conditioning (MAC) regimen (52%). The most common diagnoses that precipitated an allogeneic HSCT were acute leukemia (55%), myelodysplastic syndrome (20%), and lymphoma (13%). Significant differences in the severity of potential drug–drug interactions were not detected between genotypes among any of the six SNPs evaluated in this study (Table 1, Supplementary Table S1). Baseline demographic and clinical characteristics are detailed in Table 1.

Of the 252 patients, 250 DNA samples were obtained from the human leukocyte antigen (HLA) specimen repository at UNC McLendon Laboratories while two were collected via buccal swab. For CYP3A5*3 and CYP3A4*22, all 252 patient samples returned successful genotyping calls, but for CYP3A4*1b, there was only a sufficient quantity of high-quality DNA to return successful calls for 247 patients. When stratified by race, the allele frequencies for CYP3A5*3 and CYP3A4*1b did not significantly deviate from Hardy–Weinberg equilibrium (HWE) while CYP3A4*22 could not be properly evaluated for HWE because the predicted MAF for CYP3A4*22 is ≤ <5% among all races. For *ABCB1*, genotype calls were only obtained for 224, 187, and 222 patients that had high-quality DNA for SNPs at the C1236T, C2677T, and C3435T loci, respectively. None of the *ABCB1* SNPs deviated from HWE (Supplementary Table S2). Additionally, the predicted allele frequencies between races based on 1000 Genomes data were collected from the HaploReg v4.1 database [36,37]. These predicted allele frequencies were compared to the observed allele frequencies from this study population. Among five of the SNPs evaluated in this study, the observed and predicted allele frequencies were consistent among both black and white patients. However, the predicted allele for CYP3A5*1 for black patients was 81% while the observed allele frequency was 55% (Supplementary Table S3).

### 2.1. Association Between Steady-State Tacrolimus Trough Concentrations and CYP3A4/5 and ABCB1 SNPs

**Figure 2:**
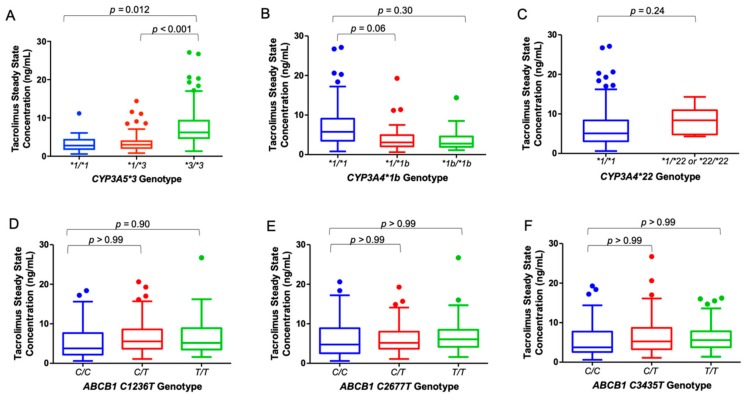
First tacrolimus trough concentration at steady-state for *CYP3A4/5* and *ABCB1* SNPs. Associations between steady-state tacrolimus trough concentrations measured on the allogeneic HSCT (Day 0) and *CYP3A4/5* (**A**–**C**) and *ABCB1* (**D**–**F**) SNPs were evaluated. Box-and-whisker plots depict median steady-state tacrolimus trough concentrations and IQR. Reported *p* values were adjusted for multiple comparisons using a Bonferroni correction. Abbreviations: ABCB1, ATP-binding cassette B1; CYP3A4/5, cytochrome P450 isoforms 4/5, IQR, inter-quartile range; SNP, single nucleotide polymorphisms.

The associations between *CYP3A4/5* SNPs and median steady-state tacrolimus trough concentrations were significant (Table 2). Specifically, CYP3A5*1/*1 patients had significantly lower median steady-state tacrolimus trough concentrations than CYP3A5*3/*3 patients (2.8 [range, 0.6–11.2] vs. 6.2 [1.3–27.1] ng/mL; *p* = 0.002). CYP3A5*1/*3 patients had significantly lower median tacrolimus trough concentrations than CYP3A5*3/*3 patients (3.0 [0.8–14.4] vs. 6.2 [1.3–27.1] ng/mL; *p* < 0.001) (Figure 2A, Table 2). After adjusting for multiple comparisons, both associations remained significant (*p* = 0.012 and *p* < 0.01, respectively). CYP3A4*1b/*1b patients trended toward lower median steady-state tacrolimus trough concentrations when compared to CYP3A4*1/*1 patients (2.8 [1.1–14.4] vs. 5.8 [0.8–27.1] ng/mL; *p* = 0.05), and CYP3A4*1/*1b patients had significantly lower trough concentrations than CYP3A4*1/*1 patients (3.1 [0.6–19.3] vs. 5.8 [0.8–27.1] ng/mL; *p* = 0.01) (Figure 2B, Table 2). However, the association between CYP3A4*1/*1b and CYP3A4*1/*1 patients was no longer significant after adjusting for the multiple comparisons. Significant differences in median steady-state tacrolimus trough concentrations were observed between CYP3A4*1/*1 patients and patients who harbored at least one CYP3A4*22 allele (5.1 [0.6–27.1] vs. 8.4 [4.3–14.3] ng/mL; *p* = 0.04) (Figure 2C, Table 2). However, again, this association was no longer significant after adjusting for the multiple comparisons. No significant differences were detected between all three *ABCB1* SNPs and the median tacrolimus trough concentration (*p* > 0.05) (Figure 2E,F, and Table 2). Last, no significant differences were detected between the severity of potential drug-drug interactions and median tacrolimus trough concentration (*p* > 0.05) (Supplementary Table S4).

### 2.2. Associations Between Steady-State Tacrolimus Trough Concentration for the First 2 Weeks Post-HSCT and CYP3A4/5 and ABCB1 SNPs

**Figure 3:**
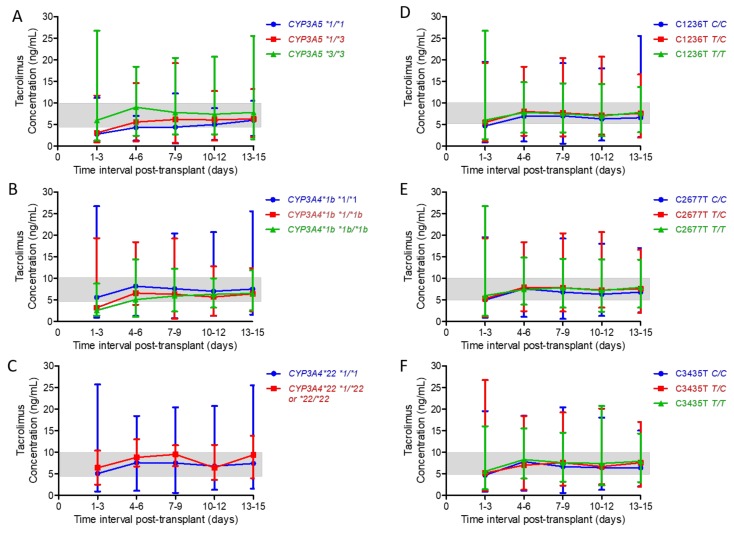
Median steady-state tacrolimus trough concentrations during the first 15 days post-transplant for *CYP3A4/5* and *ABCB1* SNPs. Associations between steady-state tacrolimus trough concentrations measured in 3-day intervals and *CYP3A4/5* (**A**–**C**) and *ABCB1* (**D**–**F**) SNPs were evaluated. The x-axis denotes time in 3-day intervals for the first 15 days post-transplant while the y-axis represents the median tacrolimus trough concentrations. The shaded area represents the goal tacrolimus trough concentration (5–10 ng/mL). Abbreviations: ABCB1, ATP-binding cassette B1; CYP3A4/5, cytochrome P450 isoforms 4 and 5; SNP, single nucleotide polymorphisms.

On average, tacrolimus TDM was obtained every 3 days from the day of the allogeneic HSCT (day 0) until day +15 post-allogeneic HSCT, as depicted below in Table 3. When compared with CYP3A5*3/*3 patients, who reached target steady-state tacrolimus trough concentrations between days +1 and +3, CYP3A5*1/*1 patients did not reach target trough concentrations until days +10–12 while CYP3A5*1/*3 patients did not reach target trough concentrations until days +4–6. In addition, significant differences in median steady-state tacrolimus trough concentrations were observed at each of the five 3-day intervals, where CYP3A5*3/*3 patients achieved higher concentrations than CYP3A5*1/*1 and CYP3A5*1/*3 patients (*p* < 0.05 for both SNPs at all intervals; Figure 3A and Table 3). Moreover, all associations between the median steady-state trough concentrations and CYP3A5*3 genotype remained significant at each 3-day interval after adjusting for multiple comparisons (*p* < 0.01; Table 3).

When compared with CYP3A4*1/*1 patients, who reached target steady-state tacrolimus trough concentrations between days +1 and +3, CYP3A4*1/*1b and CYP3A4*1b/*1b patients did not reach target trough concentrations until days +4–6. While the associations between the median steady-state trough concentrations for days +1–3 and the CYP3A4*1b genotype remained significant after adjusting for multiple comparisons (*p* < 0.01), the associations of days +4–6 median trough concentrations and the CYP3A4*1b genotype did not (*p* > 0.05; Figure 3B and Table 3). Both CYP3A4*1/*1 patients and patients who harbored at least one CYP3A4*22 allele reached target tacrolimus trough concentrations between days +1 and 3. However, significant associations were not observed between the median steady-state tacrolimus trough concentrations and this SNP at any of the five 3-day intervals (*p* > 0.05; Figure 3C and Table 3).

Analyses were also conducted for *ABCB1* SNPs. Patients with the *C/C* genotype at the *ABCB1* C1236T and C3435T loci reached target tacrolimus trough concentrations between days +4 and +6, compared with days +1 and +3 for patients with the *C/T* and *T/T* genotypes at these two loci. However, these differences were not observed to be significant (*p* > 0.05; Figure 3D,F, and Table 3). For *ABCB1* C2677T, patients with the *C/C* genotype had significantly lower target tacrolimus trough concentrations between days +10 and +12 (Figure 3E and Table 3). However, this association was no longer significant after adjusting for multiple comparisons (*p* > 0.05).

### 2.3. Associations Between Time to Target Steady-State Tacrolimus Trough Concentrations by CYP3A4/5 and ABCB1 SNPs

**Figure 4:**
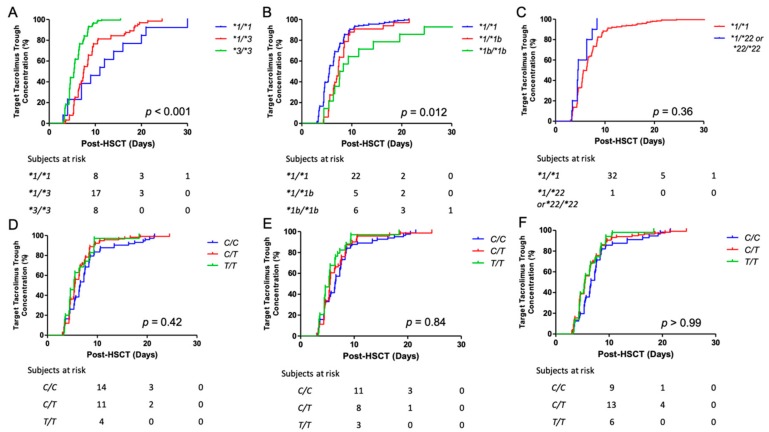
Time to target steady-state tacrolimus trough concentrations for *CYP3A4/5* and *ABCB1* SNPs. Time to target tacrolimus trough concentration, stratified by genotypes, are presented using Kaplan Meier curves, where the y-axis denotes the percentage of patients who reached a steady-state trough between 5 and 10 ng/mL while the x-axis denotes days post-transplant. Subjects at risk for not achieving tacrolimus target trough concentrations are shown on days +10, +20, and +30 post-transplant. SNPs shown below are CYP3A5*3 (**A**), CYP3A4*1b (**B**), CYP3A4*22 (**C**), *ABCB1* C1236T (**D**), *ABCB1* C2677T (**E**), and *ABCB1* C3435T (**F**). *p* < 0.05 was considered significant, and *p* values were adjusted for multiple comparisons using a Bonferroni correction. Abbreviations: ABCB1, ATP-binding cassette B1; CYP3A4/5, cytochrome P450 isoforms 4 and 5; SNP, single nucleotide polymorphisms.

Time to target trough concentration was significantly different between CYP3A5*1/*1 and *3/*3 patients (median 11.0 vs. 5.4 days; hazard ratio [HR], 0.34; 95% confidence interval [CI] 0.22–0.53; *p* = 0.004), and between CYP3A5*1/*3 and *3/*3 patients (median 7.5 vs. 5.4 days; HR, 0.39; 95% CI 0.64–2.14; *p* < 0.001). When comparing patients with CYP3A5*1/*1 or *1/*3 to patients with CYP3A5*3/*3, time to target trough concentration was also significantly different (median 7.6 vs. 5.4 days; HR, 0.36; 95% CI 0.28–0.48; *p* < 0.001). However, significant differences between CYP3A5*1/*1 and *1/*3 patients were not detected (*p* = 0.09) (Figure 4A). Time to target trough concentration was significantly different between CYP3A4*1/*1 and *1/*1b patients (median 5.5 vs. 7.3 days; HR, 1.48; 95% CI 1.01–2.07; *p* = 0.02), and between CYP3A4*1/*1 and *1b/*1b patients (median 5.5 vs. 7.9 days; HR, 2.05; 95% CI 1.33–3.16; *p* = 0.001). However, significant differences between CYP3A4*1/*1b and *1b/*1b patients were not detected (*p* = 0.08) (Figure 4B). Significant differences between CYP3A4*1/*1 and patients who harbored at least one CYP3A4*22 allele were also not detected (*p* = 0.06) (Figure 4C). Finally, for the three *ABCB1* variants, significant differences were not observed (*p* > 0.05) (Figure 4D–F).

### 2.4. Risk Factors for Supra- and Subtherapeutic Steady-State Tacrolimus Trough Concentrations

In a univariable logistic regression analysis, increased age (odds ratio [OR], 1.04; 95% CI, 1.00–1.07; *p* = 0.01), decreased weight (OR, 1.02; 95% CI, 1.00–1.03; *p* = 0.02), MAC conditioning regimen (OR, 2.73; 95% CI, 1.37–5.73; *p* = 0.004), and CYP3A5*1/*3 vs *3/*3 genotype (OR, 0.18; 95% CI, 0.05–0.62; *p* = 0.01) were significantly associated with a higher incidence of supratherapeutic steady-state tacrolimus trough concentrations on day 0. A final multivariable logistic regression model demonstrated that the CYP3A5*3/*3 genotype (*p* < 0.001), decreased weight (*p* = 0.01), and MAC regimen (*p* = 0.008) were independently associated with supratherapeutic steady-state tacrolimus trough concentrations on day 0 (Table 4).

Conversely, a second univariable logistic regression analysis identified age (OR, 0.96; 95% CI, 0.94–0.98; *p* < 0.001), black race (OR, 4.39; 95% CI, 1.81–10.68; *p* < 0.001), myeloablative conditioning (MAC; OR, 0.47; 95% CI, 0.28–0.78; *p* = 0.003), CYP3A5*1/*3 vs. *3/*3 genotype (OR, 10.51; 95% CI, 5.27–22.66; *p* < 0.001), CYP3A5*1/*1 vs. *3/*3 genotype (OR, 7.27; 95% CI, 2.13–33.39; *p* = 0.001), CYP3A4*1/*1 vs. *1/*1b genotype (OR, 0.21; 95% CI 0.09–0.48; *p* < 0.001), CYP3A4*1/*1 vs. *1b/*1b genotype (OR, 0.18; 95% CI, 0.04–0.60; *p* = 0.004), *ABCB1* C1236T *C/C* vs. *C/T* (OR, 2.08; 95% CI, 1.15–3.79; *p* = 0.02), and *ABCB1* C2677T *C/C* vs. *T/T* (OR, 2.71; 95% CI, 1.18–6.60; *p* = 0.02) as clinical, demographic, and genetic factors that were significantly associated with subtherapeutic steady-state tacrolimus trough concentrations on day 0. A final multivariable logistic regression model determined that harboring at least one CYP3A5*1 allele (*p* < 0.001), and decreased age (*p* < 0.001) were independently associated with subtherapeutic steady-state tacrolimus trough concentrations on day 0 (Table 4).

### 2.5. Associations Between Tacrolimus Pharmacodynamic Endpoints and CYP3A4/5 and ABCB1 SNPs

A total of five patients developed acute kidney injury (AKI) during the first week post-transplant, with an additional 11 patients who developed AKI during the second week post-transplant. All patients who developed AKI harbored at least one loss-of-function CYP3A5*3 allele. However, significant associations were not detected between any of the three *CYP3A4/5* SNPs or any of the three *ABCB1* SNPs and AKI (*p* > 0.05; Supplementary Table S5).

Approximately 40% of patients experienced aGVHD of any grade in the first 100 days post-allogeneic HSCT (*n* = 101). The organ most commonly affected by aGVHD was the skin (68.9%), followed by the gastrointestinal tract (30.3%) and the liver (0.8%). Significant associations between *CYP3A4/5* and *ABCB1* SNPs and cumulative incidence and severity of aGVHD (all-Grade, Grade 2+, Grade 3+) were not detected (*p* > 0.05; Supplementary Table S6). There were also no significant associations observed between *CYP3A4/5* and *ABCB1* SNPs and time to all-grade aGVHD (*p* > 0.05; Supplementary Figure S1).

## 3. Discussion

This represents the largest tacrolimus pharmacogenetics study in adult recipients of allogeneic HSCT to date. This is also the only study that enrolled white and black patients to evaluate associations between genotype and steady-state concentrations in patients solely administered oral tacrolimus for the prevention of aGVHD. The analysis of germline genetic variants in *CYP3A4/5* (tacrolimus metabolism) and *ABCB1* (tacrolimus transport) is clinically relevant because it has the potential to aid in the optimization of tacrolimus dosing for allogeneic HSCT patients. CYP3A5*3 (rs776746) has been the most extensively studied germline variant known to impact tacrolimus metabolism, and a recently published CPIC guideline recognized the role of this SNP in tacrolimus PK/PD [24]. Results from this study are important because they help to address gaps in the CPIC guideline related to *CYP3A5*-guided tacrolimus dosing and adult allogeneic HSCT, and provide additional insights into associations between additional SNPs in *CYP3A4* and *ABCB1*, and tacrolimus PK/PD.

CYP3A5*3 has been linked to both interindividual differences in tacrolimus trough concentrations and tacrolimus clearance [38,39,40]. In the solid organ transplant setting, patients who harbor at least one CYP3A5*1 allele achieved significantly lower tacrolimus trough concentrations, when compared to CYP3A5*3/*3 patients, and required 1.5–2 times the tacrolimus dose to achieve similar blood concentration levels [24]. Specifically, it has been estimated that CYP3A5*1 can explain up to 45% of the inter-patient variability in the tacrolimus dose needed to achieve target trough concentrations among renal transplant patients [25]. Moreover, in a recently published meta-analysis, renal transplant patients with at least one copy of the CYP3A5*1 allele were at higher risk of acute rejection and chronic nephrotoxicity, when compared to CYP3A5*3/*3 patients [41]. While many studies in the solid organ transplant setting have linked *CYP3A5* genetic variation to tacrolimus PK variability, PK/PD data from solid organ transplant may not adequately represent tacrolimus disposition among allogeneic HSCT patients [42].

Tacrolimus has become a cornerstone of the immunosuppressive regimen used to prevent the occurrence of aGVHD post-allogenic HSCT. Studies have shown that achieving target tacrolimus trough concentrations prior to engraftment is a significant predictor of aGVHD [8,43]. Therefore, we hypothesized that clinical and genetic factors that contribute to tacrolimus PK could ultimately impact tacrolimus PD (e.g., aGVHD incidence and severity, and treatment-emergent toxicities like AKI). CYP3A5*3 (rs776746; 6986A>G) is an intragenic SNP that results in an alternatively spliced isoform in intron 3, which alters the reading frame, produces a premature termination codon, and results in a nonfunctional CYP3A5 enzyme [24,35,44]. This SNP has garnered considerable interest clinically because it has been previously reported to independently affect tacrolimus PK/PD [11,25,40]. The major finding from this study was that patients with at least one CYP3A5*1 allele achieved lower median tacrolimus trough concentrations, and took significantly longer to achieve therapeutic concentrations, when compared to CYP3A5*3/*3 patients. Our results were consistent with data presented in the CPIC guidelines for solid organ transplant patients, which state that patients who harbor at least one CYP3A5*1 allele will achieve lower tacrolimus steady-state trough concentrations, and delayed achievement of target concentrations [24]. Our study revealed that CYP3A5*1/*1 patients required a significantly longer time to achieve tacrolimus target trough concentrations versus CYP3A5*3/*3 patients.

While we did not observe significant differences in aGVHD incidence or severity between the *CYP3A5* genotypes, it has been shown that achieving early target tacrolimus trough concentrations prior to engraftment may be important [8,43]. Because our patients all achieved steady-state tacrolimus concentrations by engraftment (generally between day +14 and +21 post-transplant), this could explain why we did not observe significant differences. Other potential reasons that we did not observe significant associations could include the retrospective study design without randomization, a heterogeneous cohort of patients, or even that despite a cohort of 252 patients we were still potentially underpowered to detect significant differences. However, importantly, approximately 40% of patients were diagnosed with aGVHD, which is consistent with previously reported estimates (35–50% aGVHD incidence among allogeneic HSCT patients) [45,46]. Therefore, a future direction of this work will be to evaluate SNPs in additional candidate genes of interest (e.g., POR*28 and *CYB5R2*) that could impact tacrolimus PK/PD [34,47,48].

CYP3A4*1b (rs2740574; −392A>G) results in an SNP located the gene promoter region. Similar to data from our study, others have found that CYP3A4*1b could be a gain-of-function variant, where patients who harbor at least one CYP3A4*1b allele required a higher tacrolimus dose to reach target trough concentrations [32,49]. However, data in the literature pertaining to the functional effect of CYP3A4*1b remain conflicting, as evidence in at least two additional studies has shown evidence of reduced enzymatic activity because of the CYP3A4*1b SNP [50,51]. Similar to *CYP3A5*, data from this study also demonstrated that patients who harbor at least one CYP3A4*1b allele achieved significantly lower steady-state tacrolimus trough concentrations than CYP3A4*1/*1 patients, and that CYP3A4*1b/*1b patients also required a significantly longer time achieve target trough concentrations than CYP3A4*1/*1 patients. CYP3A4*22 (rs35599367; 15389C>T) results in a splice variant with a 255 base pair intron 6 retention, which causes reduced production of functional full-length *CYP3A4* mRNA [52]. It has been reported that this is a loss-of-function variant, and patients with at least one CYP3A4*22 allele have achieved higher tacrolimus plasma concentrations at standard dosing [53]. In this study, patients with at least one CYP3A4*22 allele were found to have significantly higher tacrolimus concentrations at steady state than CYP3A4*1/*1 patients. These findings were consistent with previously published studies in solid organ transplant patients [54,55]. However, CYP3A4*22 was not associated with time to target tacrolimus trough concentration. Importantly, CYP3A4*1b and *22 were not significantly associated with tacrolimus PD.

Tacrolimus-induced nephrotoxicity, which manifests as AKI, is a common treatment-emergent toxicity [56]. Achieving the tacrolimus target trough is important to reduce nephrotoxicity resulting from supratherapeutic tacrolimus concentrations. Three loss-of-function germline variants located in the exonic regions of *ABCB1* (rs1128503, C1236T; rs2032582, C2677T; rs1045642, C3435T) have been implicated in both inter-patient tacrolimus PK variability and treatment-emergent toxicities [30,31,32]. In our study, AKI was not significantly associated with any of the *ABCB1* or *CYP3A4/5* SNPs. This observed lack of association can potentially be attributed to routine tacrolimus TDM and daily assessment of renal function, resulting in clinicians taking a more proactive approach to reduce AKI. However, future studies should evaluate treatment-emergent AKI through day +100 post-transplant because it can often be observed later than day +15, particularly when sulfamethoxazole/trimethoprim is added for *Pneumocystis jiroveci* prophylaxis. Alternatively, data from this study also suggest that a weight-based dosing paradigm might lead to low steady-state tacrolimus trough concentrations on day 0, which may blunt any signal of an association between SNPs and AKI.

Race was also a predictor of tacrolimus PK variability in this study, where black patients were at significantly greater risk for subtherapeutic tacrolimus trough concentrations. This observation is not surprising as data from the 1000 Genomes Project [36] estimates the CYP3A5*1 allele frequency to be approximately 70–81% among individuals from African ancestry (compared to estimates ranging from 7–37% in non-black populations). However, previous pharmacogenetics studies of tacrolimus in allogeneic HSCT failed to adequately address this allele frequency disparity, and did not enroll enough black patients to properly evaluate this public health issue [11,34]. Our study included the highest percentage of black patients (11.9%) among all pharmacogenetic studies where orally dosed tacrolimus is used in adult HSCT patients.

Oher clinical covariates that significantly associated with a higher incidence of supratherapeutic steady-state tacrolimus trough concentrations were conditioning regimen intensity and weight. One plausible explanation for the observed association between MAC and supratherapeutic concentrations is that MAC regimens can cause damage to the gut mucosal barrier [57], which can adversely affect tacrolimus absorption and gut metabolism and ultimately alter tacrolimus exposure. Alternatively, while renal clearance explains the minority of tacrolimus clearance (≤15%) [17], higher doses of methotrexate that are used for aGVHD prophylaxis in MAC regimens could affect the renal elimination of tacrolimus (particularly in patients with pre-existing renal dysfunction). However, additional studies are required to elucidate the exact relationship between MAC and supratherapeutic tacrolimus concentrations. Increased weight was identified as an independent risk factor for supratherapeutic tacrolimus trough concentrations. Increased weight has been associated with altered intestinal membrane permeability and increased paracellular transport, which all contribute to enhanced oral bioavailability [58,59], and tacrolimus oral bioavailability has been reported to be higher among overweight subjects after oral administration [59,60,61]. Additionally, decreased age has previously been implicated in suboptimal tacrolimus oral bioavailability, and in this study was identified as an independent risk factor for subtherapeutic tacrolimus trough concentrations. It has also been reported that the rate of tacrolimus metabolism decreases with age [62,63]. Thus, younger patients, with a higher metabolic potential, are likely to exhibit faster tacrolimus clearance, leading to subtherapeutic trough concentrations. This association is also consistent with a previous finding that decreased age was independently associated with higher dosing requirements and lower tacrolimus concentration/dose ratios [64].

Several limitations inherent to retrospective research were present in this study. First, we considered the effects of phenoconversion due to concomitantly prescribed strong and moderate CYP3A4/5 and P-gp inhibitors and inducers [65,66]. However, we are unable to confirm the effects of phenoconversion on tacrolimus PK/PD because the approach to tacrolimus dose adjustments due to potential drug–drug interactions with strong and moderate CYP3A4/5 and P-gp inhibitors was inconsistent among our prescribers. Second, there was a lack of standardized documentation to fully evaluate tacrolimus-induced neurotoxicity, which often manifests as tremor [67,68,69]. Therefore, in our ongoing prospective *CYP3A4/5*-guided study, we have included tacrolimus-induced toxicities, including both neurotoxicity and nephrotoxicity, using the Common Terminology Criteria for Adverse Events v5.0 criteria [70] and comprehensive medication reconciliation to monitor for new prescriptions, over-the-counter medications, and herbal supplements, as well as relevant dose changes to those medications and products.

In conclusion, this study confirmed that *CYP3A4/5* SNPs influence tacrolimus PK, and were significantly associated with both the first tacrolimus steady-state concentration and the time-to-target trough concentrations. Significant risk factors, including weight, age, black race, and conditioning regimen, were also significantly associated with supra- and/or subtherapeutic tacrolimus steady-state concentrations. These results are clinically relevant because they provide rationale to pursue future prospective lines of clinical and translational research inquiry to evaluate ways to more precisely dose tacrolimus. Precision dosing models, based on population pharmacokinetics and incorporated pharmacogenetics information, have the distinct potential to optimize tacrolimus efficacy, minimize treatment-emergent toxicities, and reduce overall healthcare expenditures.

## 4. Materials and Methods

The primary study endpoint evaluated the association between CYP3A5*3, and steady-state tacrolimus trough concentrations. The study also evaluated associations between additional SNPs in *CYP3A4* and *ABCB1* and secondary PK endpoints: Tacrolimus steady-state trough concentrations, the median tacrolimus trough concentrations for the first 2 weeks post-transplant, the time to the institutional target tacrolimus trough concentrations (5–10 ng/mL) [71,72], and the risk factors that led to supra- and subtherapeutic steady-state tacrolimus trough concentrations. Last, the study evaluated associations between SNPs in *CYP3A4/5* and *ABCB1* and secondary PD endpoints: aGVHD incidence and severity, time to aGVHD, and incidence of treatment-emergent AKI.

### 4.1. Patient Eligibility and Clinical Data Extraction

All study patients provided informed consent prior to study enrollment, and this study was approved by the University of North Carolina Institutional Review Board (UNC IRB #16-1480; approved on 05/25/2016). Inclusion criteria for this single institution pharmacogenetics study included adult patients (≥18 years of age) who were treated at University of North Carolina Medical Center (UNCMC) between January 11, 2011 and May 31, 2016, who received their first allogeneic HSCT, who were prescribed oral tacrolimus as part of their aGVHD prophylaxis regimen, and who received active follow-up at UNCMC. Patient demographic and clinical data were extracted from UNCMC’s electronic medical records (EMR; WebCis from January 1, 2011 through April 3, 2014, and Epic@UNC thereafter), and the UNC BMT Program database. For each patient, extraction of baseline clinical and demographic data began on the first date of the patient’s BMT admission (if data were not available from their admission date, data from the date closest to admission were used). Demographic data that were extracted from the EMR included patient age at the time of transplant, sex, and self-reported race. Clinical data that were collected included transplant diagnosis, date of transplant, baseline weight, baseline liver function tests, baseline serum creatinine (SCr), HLA match/mismatch, allogeneic HSCT type (matched related or unrelated donor), source of transplanted cells (PBSCs, bone marrow, or cord blood), conditioning regimen intensity (MAC versus RIC), and Karnofsky performance status score (0–100). Patient medication lists in the EMR were screened for moderate to severe drug–drug interactions (e.g., strong and moderate CYP3A4/5 or P-gp inducers and inhibitors), and the interactions were coded as categorical data (no drug–drug interaction, risk of a minimal interaction, moderate risk interaction, and risk of a severe interaction) based on drug–drug classifications accessible in LexiComp^®^Drug Interactions software [73].

Following the standard UNC institutional BMT protocol, orally administered tacrolimus was initiated on day-3 prior to allogeneic HSCT at a weight-based dose of 0.03 mg/kg twice daily. Plasma tacrolimus concentrations were considered to be at steady state by the day of transplant (day 0) based on an estimated terminal elimination tacrolimus half-life of 12 h [12,16,74]. For the UNC BMT Program, the goal tacrolimus trough concentration ranges from 5–10 ng/mL, and the first steady-state tacrolimus trough concentration was collected on day 0 after the patient received at least 5 doses. For all time-to-event analyses, tacrolimus dose, tacrolimus trough concentrations, and the date that patients achieved their first tacrolimus trough concentration of ≥ 5 ng/mL were collected.

The incidence, severity, and time to onset of aGVHD were also collected for each patient, and aGVHD was graded per the Glucksberg grading system [75,76]. For the first incidence of aGVHD, provider notes in the EMR were queried to obtain information regarding dates of onset, organs affected, and the severity of the reaction. When the clinical grade for dermatologic aGVHD was not explicitly provided, the total area of rash described in the note was used to calculate a grade. For grading hepatic aGVHD, total bilirubin changes from the date of diagnosis to date of aGVHD confirmation were used. For grading gastrointestinal aGVHD, a combination of physician-assigned initial grade, the type of aGVHD treatment the patient received, and biopsy information were all used. The date, organ, and severity of all subsequent episodes were also recorded.

For this study, nephrotoxicity was defined as an episode of AKI, and AKI was defined as an increase in SCr to greater or equal to 1.5 times baseline, per the 2012 KDIGO guidelines [77]. Tacrolimus trough concentrations obtained at a minimum interval of twice weekly during the first two weeks post-transplant (day +1–15) were used to evaluate associations between *CYP3A4/5* and *ABCB1* SNPs and nephrotoxicity.

### 4.2. Measurement of Steady-State Tacrolimus Blood Concentrations

Whole blood samples were stored at room temperature at the UNC McLendon Laboratories for two weeks from the date of collection. Tacrolimus concentrations from whole blood samples were quantified using liquid chromatography-tandem mass spectrometry, and treated with a protein precipitant reagent containing internal standard. The samples were centrifuged and chromatographed using a Waters Alliance 2795 Separations Module and Waters Xbridge C18 2.5 µM, 4.6 × 50 mm column. Tandem mass spectrometry detection was performed in multiple reaction monitoring mode, using ion transitions. The analytic measurement range was 1–40 ng/mL, while the reference range for the institutional protocol was 5–15 ng/mL, and the maximum dilution factor for sample measurement was 10×.

### 4.3. Genotyping Methods

Germline DNA was either obtained from UNC McLendon Laboratories from DNA previously collected for HLA-matching prior to their allogeneic HSCT, or directly collected from patients using an ORAcollect DX^®^ buccal swab (DNA Genotek, Ottawa, CN). For DNA obtained from UNC McLendon Laboratories, total genomic DNA (gDNA) was extracted using Promega^®^ Maxwell 16 Blood DNA Purification Kit (Promega, Madison, WI, USA). For DNA obtained from patient buccal swab samples, gDNA was extracted using Qiagen’s QiaAmp DNA Blood mini kit (Qiagen, Germantown, MD). All DNA was evaluated for quality and quantity using a QuantiFluor^®^ dsDNA system and a GloMax^®^ Discover Microplate Reader (Promega, Madison, WI). A total of six SNPs were included in this study: rs776746 (A>G, CYP3A5*3), rs274057 (A>G, CYP3A4*1b), rs35599367 (C>T, CYP3A4*22), rs1128503 (*ABCB1,* C1236T), rs2032582 (*ABCB1,* C2677T), and rs1045642 (*ABCB1,* C3435T). For the *CYP3A4/5* SNPs, genotyping was performed using TaqMan^®^ allelic discrimination assays (Applied Biosystems, Foster City, CA, USA), and were carried out according to the manufacturer’s instruction using a QuantStudio 6 Real-Time PCR System (Applied Biosystems, Foster City, CA, USA). Briefly, 120 ng of patient gDNA was loaded onto 384-well plates with TaqMan^®^ Genotyping Mastermix (Applied Biosystems) containing VIC and FAM reporter dyes. Sanger-based DNA sequencing was performed (Eurofins Genomics, Louisville, KY, USA) on a randomly selected subset of patient DNA samples (10%) to validate genotype calls and to confirm thresholds for allelic discrimination. Genotyping for the *ABCB1* SNPs was performed using molecular inversion probes (MIPs), which has been previously described in detail [78]. Briefly, a multigene MIPs-based next generation sequencing assay was developed in the UNC Center for Pharmacogenomics and Individualized Therapy, where pooled phosphorylated MIPs were hybridized to sample genomic DNA for a gap-filling and ligation reaction to circularize the MIPs and their targets. Hemo Klentaq (New England BioLabs, MA, USA) was used for the gap-fill step, and Ampligase^®^ (Lucigen, WI, USA) was used for the ligation step of the MIP capture reaction. For each sample, three 25-μL PCR reactions (18 cycles each) were completed for each MIP capture reaction using barcoded DNA oligonucleotides (IDT) for sample multiplexing. The pooled libraries were purified using AxyPrep Mag PCR clean-up kit (Axygen Scientific, Inc., CA, USA) at a ratio of 0.9.

### 4.4. Statistical Analyses

Descriptive statistics were used to summarize patient clinical and demographic characteristics, where categorical variables were summarized as counts and percentages and continuous variables were summarized as medians with ranges or IQRs, or as means with standard deviation, as appropriate. Once tacrolimus reached steady state on day 0, median trough concentrations were evaluated for each SNP using a Kruskal–Wallis test or a Mann–Whitney test, as appropriate. Thereafter, median steady-state tacrolimus trough concentration levels, in five 3-day intervals during the first 15 days post-transplant (day 0 through day +15), were evaluated for each SNP using a Kruskal–Wallis or Mann–Whitney test, as appropriate. A Chi-square of homogeneity test or Fisher’s exact test (as appropriate) was used to test associations between *CYP3A4/5, ABCB1* genotypes, and aGVHD incidence and aGVHD severity. Log rank tests with Kaplan–Meier curves were used for all univariable time-to-event analyses. Cox proportional hazards models were used to evaluate multivariable time-to-event analyses, and to derive HRs and 95% CIs. Univariable logistic regression was performed to ORs, and a multivariable model was used to identify risk factors associated with odds of non-therapeutic tacrolimus trough concentrations at steady state. Forward selection (*p* < 0.05) and backward elimination (*p* < 0.01) were employed in the development of all multivariable models. For CYP3A5*3, CYP3A4*1b, and the three *ABCB1* SNPs, an additive genetic model was assumed, while for CYP3A4*22, a dominant genetic model was assumed. HWE was evaluated using Fisher’s exact test with 1 degree of freedom, and SNP genotype calls were considered inconsistent with HWE if *p* < 1 × 10^−3^. The Bonferroni method was used to correct for multiple comparisons [79]. All statistical testing was two-sided, with an a priori significance (alpha) level of 0.05 (*p* < 0.05). Statistical analyses were performed using SAS JMP Pro software version 14.0.0 (SAS, Cary, NC, USA). GraphPad Prism^®^ version 8.2 (GraphPad Software, Inc., La Jolla, CA, USA) software was used to create figures.

## Figures and Tables

**Table 1:** Baseline clinical and demographic characteristics. Patients at our institution who received allogeneic hematopoietic stem cell transplant between January 11, 2011 and May 31, 2016 were consented, and enrolled in this tacrolimus pharmacogenetics study (*n* = 252). Associations with *p* < 0.05 are considered significant and are bolded below.

Patient Characteristics	Total (*n* = 252)	CYP3A5*1/*1 (*n* = 13)	CYP3A5*1/*3 (*n* = 64)	CYP3A5*3/*3 (*n* = 175)	*p*-Value
Age	52 (19–76)	55 (27–68)	50 (22–69)	54 (19–76)	0.09
Sex					
Male	145 (57.5%)	7 (53.8%)	32 (50%)	106 (60.6%)	0.39
Female	107 (42.5%)	6 (46.2%)	32 (50%)	69 (39.4%)	0.39
Primary diagnosis					
Aplastic Anemia	5 (2%)	1 (7.7%)	1 (1.6%)	3 (1.7%)	0.32
Acute Leukemia	139 (55.2%)	7 (53.8%)	31 (48.4%)	101 (57.7%)	0.44
Chronic Leukemia	16 (6.3%)	0 (0%)	7 (10.9%)	9 (5.1%)	0.17
Lymphoma	33 (13.1%)	2(15.4%)	10 (15.6%)	21 (12%)	0.74
MDS/MPS	56 (22.2%)	2 (15.4%)	14 (21.9%)	40 (22.9%)	0.82
Myeloma	3 (1.2%)	0 (0)	1 (1.6%)	2 (1.1%)	0.89
Donor					
MUD	165 (65.4%)	8 (61.5%)	23 (35.9%)	56 (32%)	0.09
MRD	87 (34.6%)	5 (38.5%)	41 (64.1%)	119 (68%)	0.09
Stem cell source					
PBSC	237 (94%)	11 (84.6%)	61 (95.3%)	165 (94.3%)	0.32
BM	14 (5.6%)	1 (15.4%)	3 (4.7%)	10 (5.7%)	0.90
Cord Blood	1 (0.4%)	1 (100%)	0 (0%)	0 (0%)	-
Conditioning regimen					
MAC	131 (52%)	4 (30.8%)	38 (59.4%)	89 (50.9%)	0.15
RIC	121 (48%)	9 (69.2%)	26 (40.6%)	86 (49.1%)	0.15
Race					
White	211 (83.7%)	4 (1.9%)	39 (18.5%)	168 (79.6%)	**<0.001**
Black	30 (11.9%)	7 (53.8%)	19 (29.7%)	4 (2.3%)	**<0.001**
Other	11 (4.4%)	2 (18.2%)	6 (54.5%)	3 (27.3%)	**0.01**
Drug-drug Interactions					
No interaction	19 (7.5%)	3 (23.1%)	4 (6.3%)	12 (6.9%)	0.09
Minimal risk interaction	41 (16.3%)	1 (7.7%)	11 (17.2%)	29 (16.6%)	0.69
Moderate risk interaction	183 (72.6%)	9 (69.2)	46 (71.9%)	128 (73.1%)	0.94
Severe risk interaction	9 (3.6%)	0 (0%)	3 (4.7%)	6 (3.4%)	0.70
Median IBW, kg (range)	85.1 (42.8–166.6)	80.8 (60.2–109.2)	86.6 (52.7–144.1)	85.4 (42.8–166.6)	0.15
Median tacrolimus starting daily dose, mg/dose (range)	4.0 (1.0–8.0)	4.0 (1.0–6.0)	4.0 (2.0–8.0)	5.0 (1.5–7.0)	0.55
Median tacrolimus trough concentration, ng/mL (range)	5.05 (0.6–27.1)	2.8 (0.6–11.2)	3.0 (0.8–14.4)	6.2 (1.3–27.1)	**<0.001**
Median time to tacrolimus goal trough, days (range)	6 (3–31)	11 (3–31)	8 (4–25)	5 (3–16)	**<0.001**
Liver function tests at admission					
AST (U/L)	33.0 (5–206)	33.0 (12–128)	25.5 (10–127)	26.0 (5–206)	0.46
ALT (U/L)	51 (12–209)	42 (20–103)	34.5 (14–177)	39 (11–209)	0.40
Tbili (mg/dL)	0.58 (0.1–2.5)	0.5 (0.1–1.4)	0.6 (0.2–2.5)	0.5 (0.1–2.1)	0.73
SCr (mg/dL) at admission	0.79 (0.34–1.58)	0.80 (0.4–2.0)	0.80 (0.3–1.8)	0.70 (0.3–1.6)	0.57

Abbreviations: ALT, alanine aminotransferase; AST, aspartate aminotransferase; BM, bone marrow; IDW, ideal body weight; MAC, myeloablative conditioning; MDS, myelodysplastic syndrome; MPS, mucopolysaccharidosis; MRD, match related donor; MUD, match unrelated donor; PBSC, peripheral blood stem cell; RIC, reduced-intensity conditioning; SCr, serum creatinine; Tbili, total bilirubin.

**Table 2:** Median steady-state tacrolimus trough concentrations for *CYP3A4/5* and *ABCB1* SNPs. Median steady-state tacrolimus concentration levels (ng/mL) were obtained for all patients on the day of the allogeneic HSCT (Day 0). Associations between the tacrolimus concentration and *CYP3A4/5* genotypes were evaluated. *p* < 0.05 was considered significant, and reported *p* values were adjusted for multiple comparisons using the Bonferroni method. Significant associations are bolded below. Abbreviations: HSCT, hematopoietic stem cell transplant; SNP, single nucleotide polymorphism.

SNP	Genotype	*n* (%)	Median Trough Concentration (ng/mL), Range	Unadjusted *p*-Value	Adjusted *p*-Value
CYP3A5*3 (*n* = 252)	*1/*1	13 (5.2)	2.8 (0.6–11.2)	**0.002**	**0.01**
	*1/*3	64 (25.4)	3.0 (0.8–14.4)	**< 0.001**	**< 0.001**
	*3/*3	175 (69.4)	6.2 (1.3–27.1)	Reference genotype	
CYP3A4*1b (*n* = 247)	*1/*1	200 (81.0)	5.8 (0.8–27.1)	Reference genotype	
	*1/*1b	33 (13.4)	3.1 (0.6–19.3)	**0.01**	0.06
	*1b/*1b	14 (5.6)	2.8 (1.1–14.4)	0.05	0.30
CYP3A4*22 (*n* = 252)	*1/*1	241 (95.6)	5.1 (0.6–27.1)	Reference genotype	
	*1/*22 or *22/*22	11 (4.4)	8.4 (4.3–14.3)	**0.04**	0.24
*ABCB1* C1236T (*n* = 224)	*C/C*	73 (32.6)	3.8 (0.6–18.4)	Reference genotype	
	*C/T*	116 (51.8)	5.6 (1.1–20.6)	0.19	> 0.99
	*T/T*	35 (15.6)	5.2 (1.6–26.7)	0.15	0.90
*ABCB1* C2677T (*n* = 187)	*C/C*	82 (43.9)	4.8 (0.6–20.6)	Reference genotype	
	*C/T*	71 (38.0)	5.2 (1.1–19.3)	> 0.99	> 0.99
	*T/T*	34 (18.2)	6.1 (1.6–26.7)	0.42	> 0.99
*ABCB1* C3435T (*n* = 222)	*C/C*	56 (25.2)	3.8 (0.6–19.3)	Reference genotype	
	*C/T*	116 (52.3)	5.3 (1.1–26.7)	0.42	> 0.99
	*T/T*	50 (22.5)	5.6 (1.4–16.2)	0.69	> 0.99

Abbreviations: ABCB1, ATP-binding cassette B1; CYP3A4/5, cytochrome P450 isoforms 4 and 5; SNP, single nucleotide polymorphism.

**Table 3:** Median steady-state tacrolimus trough concentrations during the first 15 days post-transplant. For each *CYP3A5* and *ABCB1* SNP, the time interval was divided into 3-day intervals because tacrolimus trough concentrations were measured every 3–4 days (on Mondays and Thursdays) during the first 15 days post-transplant. Median concentrations, with range, are reported. *p* < 0.05 was considered significant and bolded below. *p* values were adjusted for multiple comparisons using a Bonferroni correction.

CYP3A5*3 (*n* = 252)						
**Time Post-Transplant**	*1/*1 (*n* = 13)	*1/*3 (*n* = 64)		*3/*3 (*n* = 175)	**Unadjusted *p*-value**	**Adjusted *p*-value**
Day +1–3	2.8 (1.2–11.2)	3.1 (0.9–11.7)		6.0 (1.3–26.7)	**< 0.001**	**< 0.001**
Day +4–6	4.3 (1.3–7.0)	5.6 (1.1–14.6)		9.0 (2.4–18.4)	**< 0.001**	**< 0.001**
Day +7–9	4.4 (0.6–12.2)	6.2 (0.8–19.2)		7.8 (2.7–20.4)	**< 0.001**	**< 0.001**
Day +10–12	5.0 (1.4–8.8)	6.1 (1.3–12.8)		7.4 (2.7–20.7)	**< 0.001**	**< 0.001**
Day +13–15	6.0 (2.4–10.5)	6.3 (2.1–13.2)		7.8 (1.6–25.5)	**< 0.001**	**0.001**
CYP3A4*1b (*n* = 247)						
	*1/*1 (*n* = 200)	*1/*1b (*n* = 33)		*1b/*1b (*n* = 14)	**Unadjusted *p*-value**	**Adjusted *p*-value**
Day +1–3	5.6 (0.9–26.7)	3.2 (1.3–19.3)		2.6 (1.4–8.8)	**< 0.001**	**0.002**
Day +4–6	8.2 (1.3–18.4)	6.6 (3.9–18.4)		5.1 (1.1–14.4)	**0.02**	0.12
Day 7–9	7.6 (0.8–20.4)	6.3 (0.6–19.2)		5.9 (2.3–12.2)	0.08	0.48
Day +10–12	7.0 (1.3–20.7)	5.7 (1.4–12.8)		6.3 (3.2–10.0)	0.12	0.72
Day +13–15	7.5 (1.6–25.5)	6.4 (2.4–12.4)		6.5 (2.6–11.9)	0.17	0.99
CYP3A4*22 (*n* = 252)						
	*1/*1 (*n* = 241)		*1/*22 or *22/*22 (*n* = 11)		**Unadjusted *p*-value**	**Adjusted *p*-value**
Day +1–3	5.1 (0.9–25.7)		6.4 (2.5–10.4)		0.46	> 0.99
Day +4–6	7.6 (1.1–18.4)		8.8 (6.7–13.0)		0.21	> 0.99
Day +7–9	7.5 (0.6–20.4)		9.5 (6.8–11.6)		0.05	0.30
Day +10–12	6.8 (1.3–20.7)		6.4 (3.6–11.7)		0.56	> 0.99
Day +13–15	7.4 (1.6–25.5)		9.4 (4.0–13.8)		0.29	> 0.99
***ABCB1 C1236T* (*n* = 224)**						
	***C/C* (*n* = 73)**	***C/T* (*n* = 116)**		***T/T* (*n* = 35)**	**Unadjusted *p*-value**	**Adjusted *p*-value**
Day +1–3	4.7 (0.9–19.5)	5.5 (1.1–19.3)		6.0 (1.6–26.7)	0.21	> 0.99
Day +4–6	6.9 (1.1–18.4)	8.0 (2.4–18.4)		7.8 (3.1–14.8)	0.33	> 0.99
Day +7–9	7.0 (0.6–19.2)	7.7 (2.3–20.4)		7.5 (3.2–14.5)	0.45	> 0.99
Day +10–12	6.3 (1.3–18.0)	7.2 (2.7–20.7)		7.0 (2.3–14.4)	0.12	0.72
Day +13–15	6.6 (2.1–25.5)	7.6 (2.0–16.6)		7.8 (3.2–13.7)	0.66	> 0.99
***ABCB1* C2677T (*n* = 187)**						
	***C/C* (*n* = 82)**	***C/T* (*n* = 71)**		***T/T* (*n* = 34)**	**Unadjusted *p*-value**	**Adjusted *p*-value**
Day +1–3	5.0 (0.9–19.5)	5.3 (1.1–19.3)		6.0 (1.3–26.7)	0.46	> 0.99
Day +4–6	7.6 (1.1–18.4)	7.9 (2.4–18.4)		7.4 (3.9–14.8)	0.92	> 0.99
Day +7–9	6.8 (0.6–19.2)	7.8 (2.3–20.4)		7.8 (3.2–14.5)	0.13	0.78
Day +10–12	6.3 (1.3–18.0)	7.3 (3.2–20.7)		7.2 (2.3–14.4)	**0.03**	0.18
Day +13–15	6.8 (2.1–17.0)	7.6 (2.0–16.6)		7.9 (3.2–14.3)	0.34	> 0.99
***ABCB1* C3435T (*n* = 222)**						
	***C/C* (*n* = 56)**	***C/T* (*n* = 116)**		***T/T* (*n* = 50)**	**Unadjusted *p*-value**	**Adjusted *p*-value**
Day +1–3	4.7 (0.9–19.5)	5.2 (1.1–26.7)		5.6 (1.4–16.0)	0.52	> 0.99
Day +4–6	7.8 (1.1–18.4)	7.0 (1.3–18.4)		8.3 (3.9–15.5)	0.49	> 0.99
Day +7–9	6.7 (0.6–20.4)	7.6 (2.3–19.2)		7.6 (3.2–14.5)	0.56	> 0.99
Day +10–12	6.4 (1.3–18.0)	6.7 (2.6–20.1)		7.4 (2.3–20.7)	0.27	> 0.99
Day +13–15	6.4 (2.1–15.0)	7.6 (2.0–17.0)		7.9 (3.1–14.3)	0.08	0.48

Abbreviations: ABCB1, ATP-binding cassette B1; CYP3A4/5, cytochrome P450 isoforms 4/5; SNP, single nucleotide polymorphism.

**Table 4:** Analyses of supratherapeutic and subtherapeutic steady-state tacrolimus trough concentrations. Univariate logistic regression analyses evaluated supratherapeutic (>15 ng/mL) and subtherapeutic (<5 ng/mL) steady-state tacrolimus trough concentrations for clinical and demographic characteristics, as well as *CYP3A4/5* and *ABCB1* SNPs. Factors that significantly associated with supratherapeutic steady-state tacrolimus trough concentrations (*p* < 0.05) were evaluated as potential covariates in multivariable analyses. Factors that associated with significantly increased odds of supratherapeutic or subtherapeutic steady-state tacrolimus trough concentrations (*p* < 0.05) are bolded below.

Variable	Odds Ratio of Supratherapeutic Trough Concentration (95% CI)	*p*-Value	Odds Ratio of Subtherapeutic Trough Concentration (95% CI)	*p*-Value
**Univariable logistic regression analysis**				
Age (years)	1.04 (1.00–1.07)	**0.01**	0.96 (0.94–0.98)	**< 0.001**
Weight (kg)	1.02 (1.00–1.03)	**0.02**	1.00 (0.99–1.01)	0.97
Liver function tests at admission				
ALT (U/L)	0.99 (0.98–1.00)	0.07	1.00 (0.99–1.01)	0.90
AST (U/L)	0.99 (0.98–1.00)	0.05	1.00 (0.99–1.01)	0.99
Tbili (mg/dL)	1.56 (0.49–4.98)	0.44	0.69 (0.31–1.49)	0.34
SCr (mg/dL) at admission	0.93 (0.28–3.38)	0.90	1.37 (0.54–3.48)	0.51
Starting dose (mg)	0.77 (0.58–1.03)	0.07	1.13 (0.93–1.40)	0.23
Sex (female vs. male)	1.18 (0.60–2.35)	0.63	0.78 (0.47–1.28)	0.32
Race (Black vs. White)	0.74 (0.24–2.25)	0.60	4.39 (1.81–10.68)	**< 0.001**
HLA (perfect match vs. 1 or 2 mismatch)	2.05 (0.97–4.33)	0.06	0.94 (0.50–1.74)	0.83
Donor type (MRD vs. MUD)	1.33 (0.66–2.85)	0.43	0.79 (0.47–1.34)	0.39
Conditioning (MAC vs. RIC)	2.73 (1.37–5.73)	**0.004**	0.47 (0.28–0.78)	**0.003**
Stem cell source (PBSC vs. BM or cord blood)	1.31 (0.29–4.36)	0.69	0.76 (0.27–2.16)	0.60
CYP3A5*3				
*1/*1 vs. *1/*3	0.59 (0.06–6.17)	0.66	1.45 (0.29–5.70)	0.62
*1/*3 vs. *3/*3	0.18 (0.05–0.62)	**0.01**	10.51 (5.27–22.66)	**< 0.001**
*1/*1 vs. *3/*3	0.31 (0.04–2.47)	0.27	7.27 (2.13–33.39)	**0.001**
CYP3A4*1b				
*1/*1 vs. *1/*1b	2.27 (0.66–7.84)	0.19	0.21 (0.09–0.48)	**< 0.001**
*1/*1b vs. *1b/*1b	1.3 (0.12–13.70)	0.83	0.85 (0.16–3.61)	0.83
*1/*1 vs. *1b/*1b	2.95 (0.37–23.27)	0.30	0.18 (0.04–0.60)	**0.004**
CYP3A4*1/*1 vs. CYP3A4*1/*22 or *22/*22	0.77 (0.18–5.24)	0.76	2.09 (0.57–9.89)	0.27
*ABCB1* C1236T				
*C/C* vs. *C/T*	0.89 (0.40–2.00)	0.78	2.08 (1.15–3.79)	**0.02**
*C/T* vs. *T/T*	0.95 (0.35–2.59)	0.92	1.06 (0.49–2.32)	0.88
*C/C* vs. *T/T*	0.84 (0.28–2.50)	0.76	2.2 (0.98–5.08)	0.06
*ABCB1* C2677T				
*C/C* vs. *C/T*	0.90 (0.39–2.20)	0.82	1.46 (0.77–2.77)	0.25
*C/T* vs. *T/T*	0.94 (0.28–2.85)	0.92	1.86 (0.79–4.61)	0.16
*C/C* vs. *T/T*	0.85 (0.26–2.45)	0.77	2.71 (1.18–6.60)	**0.02**
*ABCB1* C3435T				
*C/C* vs. *C/T*	0.93 (0.46–2.55)	0.87	1.93 (1.01–3.70)	0.05
*C/T* vs. *T/T*	0.77 (0.30–1.96)	0.59	1.10 (0.57–2.13)	0.78
*C/C* vs. *T/T*	0.83 (0.29–2.42)	0.73	1.75 (0.81–3.79)	0.16
No DDI vs. DDI	0.96 (0.27–3.46)	0.95	1.29 (0.50–03.28)	0.60
**Multivariable logistic regression analysis of supratherapeutic trough concentrations**				
CYP3A5 *1/*1 or *1/*3 vs. *3/*3	0.43 (0.25–0.74)	**< 0.001**	-	-
Increased weight	1.02 (1.00–1.04)	**0.01**	-	-
Conditioning (MAC vs. RIC)	2.63 (1.26–5.49)	**0.008**	-	-
**Multivariable logistic regression analysis of subtherapeutic trough concentrations**				
CYP3A5 *1/*1 or *1/*3 vs. *3/*3	-	-	3.15 (2.24–4.42)	**< 0.001**
Decreased age	-	-	0.96 (0.94–0.98)	**< 0.001**

Abbreviations: ABCB1, ATP-binding cassette B1; ALT, alanine aminotransferase; AST, aspartate aminotransferase; BM, bone marrow; HLA, human leukocyte antigen; CI, confidence interval; CYP3A4/5, cytochrome P450 isoforms 4 and 5; DDI, drug–drug interaction; MAC, myeloablative conditioning; MRD, match related donor; MUD, match unrelated donor; PBSC, peripheral blood stem cell; RIC, reduced-intensity conditioning; SCr, serum creatinine; SNP, single nucleotide polymorphism.

## Supplementary Materials

Supplementary materials can be found at https://www.mdpi.com/1422-0067/21/3/858/s1.

## References

[1] Baron F., Storb R. (2004). Allogeneic hematopoietic cell transplantation as treatment for hematological malignancies: A review. Springer Semin. Immunopathol..

[2] Peccatori J., Ciceri F. (2010). Allogeneic stem cell transplantation for acute myeloid leukemia. Haematologica.

[3] Ferrara J.L.M., Levine J.E., Reddy P., Holler E. (2009). Graft-versus-host disease. Lancet.

[4] Moon J.H., Kim S.N., Kang B.W., Chae Y.S., Kim J.G., Ahn J.S., Kim Y.K., Yang D.H., Lee J.J., Kim H.J. (2010). Early onset of acute GVHD indicates worse outcome in terms of severity of chronic GVHD compared with late onset. Bone Marrow Transplant..

[5] Khoury H.J., Wang T., Hemmer M.T., Couriel D., Alousi A., Cutler C., Aljurf M., Antin J.H., Ayas M., Battiwalla M. (2017). Improved survival after acute graft-versus-host disease diagnosis in the modern era. Haematologica.

[6] van Gelder T., Hesselink D.A. (2010). Dosing tacrolimus based on CYP3A5 genotype: Will it improve clinical outcome?. Clin. Pharmacol. Ther..

[7] Wallin E.F., Hill D.L., Linterman M.A., Wood K.J. (2018). The calcineurin inhibitor tacrolimus specifically suppresses human T follicular helper cells. Front. Immunol..

[8] Ganetsky A., Shah A., Miano T.A., Hwang W.T., He J., Loren A.W., Hexner E.O., Frey N.V., Porter D.L., Reshef R. (2016). Higher tacrolimus concentrations early after transplant reduce the risk of acute GvHD in reduced-intensity allogeneic stem cell transplantation. Bone Marrow Transplant..

[9] Sikma M.A., Hunault C.C., van de Graaf E.A., Verhaar M.C., Kesecioglu J., de Lange D.W., Meulenbelt J. (2017). High tacrolimus blood concentrations early after lung transplantation and the risk of kidney injury. Eur. J. Clin. Pharmacol..

[10] Sikm M.A., Hunault C.C., Kirkels J.H., Verhaar M.C., Kesecioglu J., de Lange D.W. (2018). Association of Whole Blood Tacrolimus Concentrations with Kidney Injury in Heart Transplantation Patients. Eur. J. Drug Metab. Pharmacokinet..

[11] Khaled S.K., Palmer J.M., Herzog J., Stiller T., Tsai N.-C., Senitzer D., Liu X., Thomas S.H., Shayani S., Weitzel J. (2016). Influence of Absorption, Distribution, Metabolism, and Excretion Genomic Variants on Tacrolimus/Sirolimus Blood Levels and Graft-versus-Host Disease after Allogeneic Hematopoietic Cell Transplantation. Biol. Blood Marrow Transplant..

[12] Venkataramanan R., Swaminathan A., Prasad T., Jain A., Zuckerman S., Warty V., McMichael J., Lever J., Burckart G., Starzl T. (1995). Clinical pharmacokinetics of tacrolimus. Clin. Pharmacokinet..

[13] Tron C., Lemaitre F., Verstuyft C., Petitcollin A., Verdier M.-C., Bellissant E. (2019). Pharmacogenetics of membrane transporters of tacrolimus in solid organ transplantation. Clin. Pharmacokinet..

[14] Borra L.C.P., Roodnat J.I., Kal J.A., Mathot R.A.A., Weimar W., van Gelder T. (2010). High within-patient variability in the clearance of tacrolimus is a risk factor for poor long-term outcome after kidney transplantation. Nephrol. Dial. Transplant..

[15] Undre N.A. (2003). Pharmacokinetics of tacrolimus-based combination therapies. Nephrol. Dial. Transplant..

[16] Wallemacq P.E., Verbeeck R.K. (2001). Comparative clinical pharmacokinetics of tacrolimus in paediatric and adult patients. Clin. Pharmacokinet..

[17] Möller A., Iwasaki K., Kawamura A., Teramura Y., Shiraga T., Hata T., Schäfer A., Undre N.A. (1999). The disposition of 14C-labeled tacrolimus after intravenous and oral administration in healthy human subjects. Drug Metab. Dispos..

[18] Dai Y., Hebert M.F., Isoherranen N., Davis C.L., Marsh C., Shen D.D., Thummel K.E. (2006). Effect of CYP3A5 polymorphism on tacrolimus metabolic clearance in vitro. Drug Metab. Dispos..

[19] Hebert M.F. (1997). Contributions of hepatic and intestinal metabolism and P-glycoprotein to cyclosporine and tacrolimus oral drug delivery. Adv. Drug Deliv. Rev..

[20] Anglicheau D., Verstuyft C., Laurent-Puig P., Becquemont L., Schlageter M.-H., Cassinat B., Beaune P., Legendre C., Thervet E. (2003). Association of the multidrug resistance-1 gene single-nucleotide polymorphisms with the tacrolimus dose requirements in renal transplant recipients. J. Am. Soc. Nephrol..

[21] Staatz C.E., Tett S.E. (2004). Clinical pharmacokinetics and pharmacodynamics of tacrolimus in solid organ transplantation. Clin. Pharmacokinet..

[22] Hesselink D.A., Bouamar R., Elens L., van Schaik R.H.N., van Gelder T. (2014). The role of pharmacogenetics in the disposition of and response to tacrolimus in solid organ transplantation. Clin. Pharmacokinet..

[23] Provenzani A., Santeusanio A., Mathis E., Notarbartolo M., Labbozzetta M., Poma P., Provenzani A., Polidori C., Vizzini G., Polidori P. (2013). Pharmacogenetic considerations for optimizing tacrolimus dosing in liver and kidney transplant patients. World J. Gastroenterol..

[24] Birdwell K.A., Decker B., Barbarino J.M., Peterson J.F., Stein C.M., Sadee W., Wang D., Vinks A.A., He Y., Swen J.J. (2015). Clinical pharmacogenetics implementation consortium (CPIC) guidelines for CYP3A5 genotype and tacrolimus dosing. Clin. Pharmacol. Ther..

[25] Haufroid V., Mourad M., Van Kerckhove V., Wawrzyniak J., De Meyer M., Eddour D.C., Malaise J., Lison D., Squifflet J.P., Wallemacq P. (2004). The effect of CYP3A5 and MDR1 (ABCB1) polymorphisms on cyclosporine and tacrolimus dose requirements and trough blood levels in stable renal transplant patients. Pharmacogenetics.

[26] Press R.R., Ploeger B.A., den Hartigh J., van der Straaten T., van Pelt J., Danhof M., de Fijter J.W., Guchelaar H.J. (2009). Explaining variability in tacrolimus pharmacokinetics to optimize early exposure in adult kidney transplant recipients. Ther. Drug Monit..

[27] Abdel-Kahaar E., Winter S., Tremmel R., Schaeffeler E., Olbricht C.J., Wieland E., Schwab M., Shipkova M., Jaeger S.U. (2019). The impact of CYP3A4*22 on tacrolimus pharmacokinetics and outcome in clinical practice at a single kidney transplant center. Front. Genet..

[28] Oetting W.S., Wu B., Schladt D.P., Guan W., Remmel R.P., Dorr C., Mannon R.B., Matas A.J., Israni A.K., Jacobson P.A. (2018). Attempted validation of 44 reported SNPs associated with tacrolimus troughs in a cohort of kidney allograft recipients. Pharmacogenomics.

[29] Shugarts S., Benet L.Z. (2009). The role of transporters in the pharmacokinetics of orally administered drugs. Pharm. Res..

[30] Hamadeh I.S., Zhang Q., Steuerwald N., Hamilton A., Druhan L.J., McSwain M., Diez Y., Rusin S., Han Y., Symanowski J. (2019). Effect of CYP3A4, CYP3A5, and ABCB1 polymorphisms on intravenous tacrolimus exposure and adverse events in adult allogeneic stem cell transplant patients. Biol. Blood Marrow Transplant..

[31] Hodges L.M., Markova S.M., Chinn L.W., Gow J.M., Kroetz D.L., Klein T.E., Altman R.B. (2011). Very important pharmacogene summary: ABCB1 (MDR1, P-glycoprotein). Pharmacogenet. Genomics..

[32] Hesselink D.A., van Schaik R.H.N., van der Heiden I.P., van der Werf M., Gregoor P.J.H.S., Lindemans J., Weimar W., van Gelder T. (2003). Genetic polymorphisms of the CYP3A4, CYP3A5, and MDR-1 genes and pharmacokinetics of the calcineurin inhibitors cyclosporine and tacrolimus. Clin. Pharmacol. Ther..

[33] Onizuka M., Kunii N., Toyosaki M., Machida S., Ohgiya D., Ogawa Y., Kawada H., Inoko H., Ando K. (2011). Cytochrome P450 genetic polymorphisms influence the serum concentration of calcineurin inhibitors in allogeneic hematopoietic SCT recipients. Bone Marrow Transplant..

[34] Suetsugu K., Mori Y., Yamamoto N., Shigematsu T., Miyamoto T., Egashira N., Akashi K., Masuda S. (2019). Impact of CYP3A5, POR, and CYP2C19 polymorphisms on trough concentration to dose ratio of tacrolimus in allogeneic hematopoietic stem cell transplantation. Int. J. Mol. Sci..

[35] Lamba J., Hebert J.M., Schuetz E.G., Klein T.E., Altman R.B. (2012). PharmGKB summary: Very important pharmacogene information for CYP3A5. Pharmacogenet. Genomics..

[36] 1000 Genomes Project Consortium (2015). A global reference for human genetic variation. Nature.

[37] Ward L.D., Kellis M. (2012). HaploReg: A resource for exploring chromatin states, conservation, and regulatory motif alterations within sets of genetically linked variants. Nucleic Acids Res..

[38] Macphee I.A.M., Fredericks S., Tai T., Syrris P., Carter N.D., Johnston A., Goldberg L., Holt D.W. (2002). Tacrolimus pharmacogenetics: Polymorphisms associated with expression of cytochrome p4503A5 and P-glycoprotein correlate with dose requirement. Transplantation.

[39] Macphee I.A.M., Fredericks S., Mohamed M., Moreton M., Carter N.D., Johnston A., Goldberg L., Holt D.W. (2005). Tacrolimus pharmacogenetics: The CYP3A5*1 allele predicts low dose-normalized tacrolimus blood concentrations in whites and South Asians. Transplantation.

[40] Quteineh L., Verstuyft C., Furlan V., Durrbach A., Letierce A., Ferlicot S., Taburet A.M., Charpentier B., Becquemont L. (2008). Influence of CYP3A5 genetic polymorphism on tacrolimus daily dose requirements and acute rejection in renal graft recipients. Basic Clin. Pharmacol. Toxicol..

[41] Rojas L., Neumann I., Herrero M.J., Bosó V., Reig J., Poveda J.L., Megías J., Bea S., Aliño S.F. (2015). Effect of CYP3A5*3 on kidney transplant recipients treated with tacrolimus: A systematic review and meta-analysis of observational studies. Pharmacogenomics J..

[42] Jacobson P., Ng J., Ratanatharathorn V., Uberti J., Brundage R.C. (2001). Factors affecting the pharmacokinetics of tacrolimus (FK506) in hematopoietic cell transplant (HCT) patients. Bone Marrow Transplant..

[43] Mori T., Kato J., Shimizu T., Aisa Y., Nakazato T., Yamane A., Ono Y., Kunimoto H., Okamoto S. (2012). Effect of early posttransplantation tacrolimus concentration on the development of acute graft-versus-host disease after allogeneic hematopoietic stem cell transplantation from unrelated donors. Biol. Blood Marrow Transplant..

[44] Staatz C.E., Goodman L.K., Tett S.E. (2010). Effect of CYP3A and ABCB1 single nucleotide polymorphisms on the pharmacokinetics and pharmacodynamics of calcineurin inhibitors: Part II. Clin. Pharmacokinet..

[45] Jagasia M., Arora M., Flowers M.E.D., Chao N.J., McCarthy P.L., Cutler C.S., Urbano-Ispizua A., Pavletic S.Z., Haagenson M.D., Zhang M.-J. (2012). Risk factors for acute GVHD and survival after hematopoietic cell transplantation. Blood.

[46] Zeiser R., Blazar B.R. (2017). Acute Graft-versus-Host Disease—Biologic Process, Prevention, and Therapy. N. Engl. J. Med..

[47] Elens L., Hesselink D.A., Bouamar R., Budde K., de Fijter J.W., De Meyer M., Mourad M., Kuypers D.R., Haufroid V., van Gelder T. (2014). Impact of POR*28 on the pharmacokinetics of tacrolimus and cyclosporine A in renal transplant patients. Ther. Drug Monit..

[48] Dorr C.R., Wu B., Remmel R.P., Muthusamy A., Schladt D.P., Abrahante J.E., Guan W., Mannon R.B., Matas A.J., Oetting W.S. (2019). Identification of genetic variants associated with tacrolimus metabolism in kidney transplant recipients by extreme phenotype sampling and next generation sequencing. Pharmacogenomics J..

[49] Shi W.-L., Tang H.-L., Zhai S.-D. (2015). Effects of the CYP3A4*1B Genetic Polymorphism on the Pharmacokinetics of Tacrolimus in Adult Renal Transplant Recipients: A Meta-Analysis. PLoS ONE.

[50] Rodríguez-Antona C., Sayi J.G., Gustafsson L.L., Bertilsson L., Ingelman-Sundberg M. (2005). Phenotype-genotype variability in the human CYP3A locus as assessed by the probe drug quinine and analyses of variant CYP3A4 alleles. Biochem. Biophys. Res. Commun..

[51] Zanger U.M., Schwab M. (2013). Cytochrome P450 enzymes in drug metabolism: Regulation of gene expression, enzyme activities, and impact of genetic variation. Pharmacol. Ther..

[52] Wang D., Sadee W. (2016). CYP3A4 intronic SNP rs35599367 (CYP3A4*22) alters RNA splicing. Pharmacogenet. Genomics.

[53] Elens L., Capron A., van Schaik R.H.N., De Meyer M., De Pauw L., Eddour D.C., Latinne D., Wallemacq P., Mourad M., Haufroid V. (2013). Impact of CYP3A4*22 allele on tacrolimus pharmacokinetics in early period after renal transplantation: Toward updated genotype-based dosage guidelines. Ther. Drug. Monit..

[54] Gervasini G., Garcia M., Macias R.M., Cubero J.J., Caravaca F., Benitez J. (2012). Impact of genetic polymorphisms on tacrolimus pharmacokinetics and the clinical outcome of renal transplantation. Transpl. Int..

[55] Tang J.T., Andrews L.M., van Gelder T., Shi Y.Y., van Schaik R.H.N., Wang L.L., Hesselink D.A. (2016). Pharmacogenetic aspects of the use of tacrolimus in renal transplantation: Recent developments and ethnic considerations. Expert Opin. Drug Metab. Toxicol..

[56] Piñana J.L., Perez-Pitarch A., Garcia-Cadenas I., Barba P., Hernandez-Boluda J.C., Esquirol A., Fox M.L., Terol M.J., Queraltó J.M., Vima J. (2017). A Time-to-Event Model for Acute Kidney Injury after Reduced-Intensity Conditioning Stem Cell Transplantation Using a Tacrolimus- and Sirolimus-based Graft-versus-Host Disease Prophylaxis. Biol. Blood Marrow Transplant..

[57] Johansson J.E., Brune M., Ekman T. (2001). The gut mucosa barrier is preserved during allogeneic, haemopoietic stem cell transplantation with reduced intensity conditioning. Bone Marrow Transplant..

[58] Teixeira T.F.S., Souza N.C.S., Chiarello P.G., Franceschini S.C.C., Bressan J., Ferreira C.L.L.F., Peluzio M.d.C.G. (2012). Intestinal permeability parameters in obese patients are correlated with metabolic syndrome risk factors. Clin. Nutr..

[59] Andrews L.M., de Winter B.C.M., Tang J.-T., Shuker N., Bouamar R., van Schaik R.H.N., Koch B.C.P., van Gelder T., Hesselink D.A. (2017). Overweight Kidney Transplant Recipients Are at Risk of Being Overdosed Following Standard Bodyweight-Based Tacrolimus Starting Dose. Transplant. Direct.

[60] Sawamoto K., Huong T.T., Sugimoto N., Mizutani Y., Sai Y., Miyamoto K. (2014). Mechanisms of lower maintenance dose of tacrolimus in obese patients. Drug Metab. Pharmacokinet..

[61] Thishya K., Vattam K.K., Naushad S.M., Raju S.B., Kutala V.K. (2018). Artificial neural network model for predicting the bioavailability of tacrolimus in patients with renal transplantation. PLoS ONE.

[62] Schütte-Nütgen K., Thölking G., Steinke J., Pavenstädt H., Schmidt R., Suwelack B., Reuter S. (2019). Fast tac metabolizers at risk—it is time for a C/D ratio calculation. J. Clin. Med..

[63] Schütte-Nütgen K., Thölking G., Steinke J., Pavenstädt H., Schmidt R., Suwelack B., Reuter S. (2019). Correction: Fast Tac Metabolizers at Risk—It is Time for a C/D Ratio Calculation. J. Clin. Med..

[64] Gijsen V., Mital S., van Schaik R.H., Soldin O.P., Soldin S.J., van der Heiden I.P., Nulman I., Koren G., de Wildt S.N. (2011). Age and CYP3A5 genotype affect tacrolimus dosing requirements after transplant in pediatric heart recipients. J. Heart Lung Transplant..

[65] Shah R.R., Smith R.L. (2015). Addressing phenoconversion: The Achilles’ heel of personalized medicine. Br. J. Clin. Pharmacol..

[66] de Leon J. (2015). Phenoconversion and therapeutic drug monitoring. Br. J. Clin. Pharmacol..

[67] Erro R., Bacchin R., Magrinelli F., Tomei P., Geroin C., Squintani G., Lupo A., Zaza G., Tinazzi M. (2018). Tremor induced by Calcineurin inhibitor immunosuppression: A single-centre observational study in kidney transplanted patients. J. Neurol..

[68] Wu G., Weng F.L., Balaraman V. (2013). Tacrolimus-induced encephalopathy and polyneuropathy in a renal transplant recipient. BMJ Case Rep..

[69] Eidelman B.H., Abu-Elmagd K., Wilson J., Fung J.J., Alessiani M., Jain A., Takaya S., Todo S.N., Tzakis A., Van Thiel D. (1991). Neurologic complications of FK 506. Transplant Proc..

[70] (2017). Common Terminology Criteria for Adverse Events (CTCAE) Version 5.0 [Internet]. National Cancer Institute: Division of Cancer Treatment & Diagnosis. https://ctep.cancer.gov/protocolDevelopment/electronic_applications/docs/CTCAE_v5_Quick_Reference_5x7.pdf.

[71] Przepiorka D., Nash R.A., Wingard J.R., Zhu J., Maher R.M., Fitzsimmons W.E., Fay J.W. (1999). Relationship of tacrolimus whole blood levels to efficacy and safety outcomes after unrelated donor marrow transplantation. Biol. Blood Marrow Transplant..

[72] Przepiorka D., Khouri I., Ippoliti C., Ueno N.T., Mehra R., Körbling M., Giralt S., Gajewski J., Fischer H., Donato M. (1999). Tacrolimus and minidose methotrexate for prevention of acute graft-versus-host disease after HLA-mismatched marrow or blood stem cell transplantation. Bone Marrow Transplant..

[73] Tacrolimus. Lexi-Drugs Online.

[74] Sikma M.A., van Maarseveen E.M., van de Graaf E.A., Kirkels J.H., Verhaar M.C., Donker D.W., Kesecioglu J., Meulenbelt J. (2015). Pharmacokinetics and toxicity of tacrolimus early after heart and lung transplantation. Am. J. Transplant..

[75] Rowlings P.A., Przepiorka D., Klein J.P., Gale R.P., Passweg J.R., Henslee-Downey P.J., Cahn J.Y., Calderwood S., Gratwohl A., Socié G. (1997). IBMTR Severity Index for grading acute graft-versus-host disease: Retrospective comparison with Glucksberg grade. Br. J. Haematol..

[76] Glucksberg H., Storb R., Fefer A., Buckner C.D., Neiman P.E., Clift R.A., Lerner K.G., Thomas E.D. (1974). Clinical manifestations of graft-versus-host disease in human recipients of marrow from HL-A-matched sibling donors. Transplantation.

[77] Khwaja A. (2012). KDIGO clinical practice guidelines for acute kidney injury. Nephron Clin. Pract..

[78] Suzuki O., Dong O.M., Howard R.M., Wiltshire T. (2019). Characterizing the pharmacogenome using molecular inversion probes for targeted next-generation sequencing. Pharmacogenomics.

[79] Bland J.M., Altman D.G. (1995). Multiple significance tests: The Bonferroni method. BMJ.

