# Supplementary material for: Influence of Germline Genetics on Tacrolimus Pharmacokinetics and Pharmacodynamics in Allogeneic Hematopoietic Stem Cell Transplant Patients
Source: Int J Mol Sci. 2020 Jan 29;21(3):858. doi: 10.3390/ijms21030858 (PMC7037631; doi:10.3390/ijms21030858)
Supplement: Supplementary file 1 [file ijms-21-00858-s001.pdf]

Supplementary Tables

Table S1. Preliminary results for association between DDIs and CYP3A4/5 and ABCB1 SNPs. The presence of one or more DDIs were categorized as one group and associations were examined or each SNP. $p < 0.05$ was considered significant.

SNP	Genotype	DDI, *n*	DDI, %	Unadjusted *p*-value	Adjusted *p*-value
*CYP3A5*3* (*n* = 252)	**1/*1* (*n* = 13)	10	76.9	0.09	0.54
	**1/*3 (n = 64)*	60	93.8		
	**3/*3* (*n* = 175)	163	93.1		
*CYP3A4*1b* (*n* = 247)	**1/*1* (*n* = 200)	189	94.5	0.08	0.48
	**1/*1b (n = 33)*	28	84.9		
	**1b/*1b (n = 14)*	12	85.7		
*CYP3A4*22* (*n* = 252)	**1/*1* (*n* = 241)	222	92.1	0.36	> 0.99
	**1/*22 or *22/*22* (*n* = 11)	10	90.9		
ABCB1 C1236T (*n* = 224)	*CC* (*n* = 73)	72	98.6	0.10	0.60
	CT (*n* = 116)	103	88.8		
	TT (*n* = 35)	31	88.6		
ABCB1 C2677T (*n* = 187)	*CC* (*n* = 82)	80	97.6	0.48	>0.99
	CT (*n* = 71)	66	93.0		
	TT (*n* = 34)	31	91.2		
ABCB1 C3435T (*n* = 222)	*CC* (*n* = 56)	55	98.2	0.11	0.66
	CT (*n* = 116)	104	89.7		
	TT (*n* = 50)	46	92.0		

Abbreviations: ABCB1, ATP-binding cassette B1; CYP3A4/5, cytochrome P450 isoforms 4 and 5; DDI, drug-drug interactions.

Table S2. HWE for *CYP3A5*3*, *CYP3A4*1b* and *ABCB1* SNPs. For *CYP3A5*3*, successful genotyping calls were obtained for all 252 patients, but for *CYP3A4*1b* there was only sufficient quantity of high-quality DNA to genotype 247 patients. Similarly, for *ABCB1*, only 224, 187, and 222 patients were genotyped for SNPs located at the C1236T, C2677T and C3435T loci, respectively. Based on predicted MAF differences among black and non-black patients for *CYP3A5*3* and *CYP3A4*1b*, subgroup analyses were performed. For the subgroup HWE analyses that were stratified by race, there were a total of 30 black patients and 222 non-black patients for *CYP3A5*3*, while there were a total of 29 black patients and 218 non-black patients for *CYP3A5*1b*. HWE calculations were not performed for *CYP3A4*22* because the predicted MAF was ≤5% among all races. SNP genotype calls were considered inconsistent with HWE only when $p < 1 \times 10^{-3}$.

	Observed Values	Expected Values	χ2	*p*-value
**HWE analysis among all patients (*n* = 252)**				
		***CYP3A5*3* (*n* = 252)**		
**1/*1*	13	8		
**1/*3*	64	74	4.55	0.03
**3/*3*	175	170		
		***CYP3A4*1b* (*n* = 247)**		
**1/*1*	200	190		
**1/*1b*	33	54	36.20	< 0.001
**1b/*1b*	14	4		
		***ABCB1 C1236T* (*n* = 224)**		
C/C	73	77		
C/T	116	109	0.99	0.32
T/T	35	39		
		***ABCB1 C2677T* (*n* = 187)**		
C/C	82	74		
C/T	71	87	6.54	0.01
T/T	34	26		
		***ABCB1 C3435T* (*n* = 222)**		
C/C	56	59		
C/T	116	111	0.47	0.49
T/T	50	53		
**HWE analysis among Black patients only (*n* = 30)**				
		***CYP3A5*3* (*n* = 30)**		
**1/*1*	7	9		
**1/*3*	19	15	2.34	0.13
**3/*3*	4	6		
		***CYP3A4*1b* (*n* = 29)**		
**1/*1*	4	3		
**1/*1b*	12	13	0.21	0.65
**1b/*1b*	13	12		
**HWE analysis among non-Black patients (*n* = 222)**				
		***CYP3A5*3* (*n* = 222)**		
**1/*1*	6	4		
**1/*3*	45	50	1.97	0.16
**3/*3*	171	169		
		***CYP3A4*1b* (*n* = 218)**		
**1/*1*	196	196		
**1/*1b*	21	22	0.28	0.59
**1b/*1b*	1	1		

Abbreviations: ABCB1, ATP-binding cassette B1; Adj, adjusted; CYP3A4/5, cytochrome P450 isoforms 4 and 5; HWE, Hardy-Weinberg equilibrium; MAF, minor allele frequency; SNP, single nucleotide polymorphisms.

Table S3. Predicted and observed minor allele frequency for *CYP3A4/5* and *ABCB1* SNPs. Predicted allele frequencies were based on 1000 Genomes data, and were obtained from the HaploReg v4.1 database that is hosted by the Broad Institute and the Massachusetts Institute of Technology (https://pubs.broadinstitute.org/mammals/haploreg/haploreg.php).

SNP	Self-Reported Race	Predicted Allele Frequency	Observed Allele Frequency
*CYP3A5*1*	Black	0.81	0.55
	White	0.05	0.11
*CYP3A4*1b*	Black	0.76	0.66
	White	0.03	0.05
*CYP3A4*22*	Black	N/A	N/A
	White	0.05	0.03
ABCB1 C1236T	Black	0.14	0.16
	White	0.43	0.44
ABCB1 C2677T	Black	0.03	0.06
	White	0.43	0.42
ABCB1 C3435T	Black	0.15	0.19
	White	0.53	0.53

Abbreviations: ABCB1, ATP-binding cassette B1; CYP3A4/5, cytochrome P450 isoforms 4 and 5; SNP, single nucleotide polymorphism.

Table S4. Preliminary results for association between DDIs and median tacrolimus steady-state trough concentrations. Median steady-state tacrolimus concentration levels (ng/mL) were obtained for all patients on the day of allogeneic HSCT (Day 0). Associations between tacrolimus concentration and different risks of DDI were evaluated. $p < 0.05$ was considered significant.

**DDI**	***n* (%)**	**Median Trough Concentration (ng/mL), Range**	***p*-value**
No interaction	19 (7.5)	4.1 (1.4–14.1)	Reference
Minimal risk of interaction	41 (16.3)	6.4 (1.1–20.3)	0.24
Moderate risk of interaction	183 (72.6)	5.0 (0.6–27.1)	0.79
Severe risk of interaction	9 (3.6)	8.8 (2.1–16.2)	0.11

Abbreviations: ABCB1, ATP-binding cassette B1; Adj, adjusted; CYP3A4/5, cytochrome P450 isoforms 4 and 5; DDI, drug-drug interaction.

Table S5. AKI occurrence for *CYP3A4/5* and *ABCB1* SNPs. Each *CYP3A4/5* variant are presented in table below with total of patients presenting AKI during the first 15 days post-allogeneic HSCT. $p < 0.05$ was considered significant, and reported *p* values were adjusted for multiple comparisons using the Bonferroni method.

SNP	Genotype	AKI, *n*	AKI, %	Unadjusted *p*-value	Adjusted *p*-value
*CYP3A5*3* (*n* = 252)	**1/*1 or *1/*3* (*n* = 77)	6	7.80	0.58	> 0.99
	**3/*3* (*n* = 175)	10	5.71		
*CYP3A4*1b* (*n* = 247)	**1/*1* (*n* = 200)	10	5.00	0.09	0.54
	**1/*1b or *1b/*1b* (*n* = 47)	6	12.77		
*CYP3A4*22* (*n* = 252)	**1/*1* (*n* = 241)	16	6.63	> 0.99	> 0.99
	**1/*22 or *22/*22* (*n* = 11)	0	0.00		
ABCB1 C1236T (*n* = 224)	*CC* (*n* = 73)	6	8.2	0.54	> 0.99
	CT (*n* = 116)	7	6.0		
	TT (*n* = 35)	1	2.9		
ABCB1 C2677T (*n* = 187)	*CC* (*n* = 82)	9	11.0	0.16	0.96
	CT (*n* = 71)	3	4.2		
	TT (*n* = 34)	1	2.9		
ABCB1 C3435T (*n* = 222)	*CC* (*n* = 56)	4	7.1	0.96	> 0.99
	CT (*n* = 116)	7	6.0		
	TT (*n* = 50)	3	6.0		

Abbreviations: ABCB1, ATP-binding cassette B1; AKI, acute kidney injury; CYP3A4/5, cytochrome P450 isoforms 4 and 5; SNP, single nucleotide polymorphism.

Table S6. Cumulative incidence of aGVHD by *CYP3A4/5* and *ABCB1*. Cumulative incidence of aGVHD occurrence, regardless of organ type, are presented below. Results are stratified by CYP3A4/5 and *ABCB1*, and grouped by all-grade, grade 2+, and grade 3+ aGVHD. $p < 0.05$ was considered significant.

	aGVHD					
**Genotype**	**All Grade** *n* **(%)**	***p*-value**	**Grade 2+** *n* **(%)**	***p*-value**	**Grade 3+** *n* **(%)**	***p*-value**
***CYP3A5*3* (n=252)**						
**1/*1* (*n* = 13)	4 (30.8)		0 (0)		0 (0)	
**1/*3* (*n* = 64)	27 (42.2)	> 0.99	11 (17.2)	> 0.99	2 (3.1)	> 0.99
**3/*3* (*n* = 175)	70 (40.0)		24 (13.7)		6 (3.4)	
***CYP3A4*1b* (n=247)**						
**1/*1* (*n* = 200)	85 (42.5)		28 (14)		7 (3.5)	
**1/*1b* (*n* = 33)	11 (33.3)	> 0.99	4 (12.1)	> 0.99	1 (3.0)	> 0.99
**1b/*1b* (*n* = 14)	3 (21.4)		1 (7.1)		0 (0)	
***CYP3A4*22* (n=252)**						
**1/*1* (*n* = 241)	99 (41.1)	> 0.99	34 (14.1)	> 0.99	8 (3.3)	> 0.99
**1/*22 or *22/*22* (*n* = 11)	2 (18.2)		0 (0)		0 (0)	
***ABCB1* C1236T (n=224)**						
C/C (*n* = 73)	38 (52.1)		13 (17.8)		0 (0)	
C/T (*n* = 116)	42 (36.2)	0.54	13 (11.2)	> 0.99	4 (3.4)	0.30
T/T (*n* = 35)	15 (42.9)		7 (20.0)		3 (8.6)	
***ABCB1* C2677T (n=187)**						
C/C (*n* = 82)	40 (48.8)		12 (14.6)		2 (2.4)	
C/T (*n* = 71)	29 (40.8)	> 0.99	9 (12.7)	> 0.99	3 (4.2)	> 0.99
T/T (*n* = 34)	14 (41.2)		5 (14.7)		1 (2.9)	
***ABCB1* C3435T (n=222)**						
C/C (*n* = 56)	27 (48.2)		10 (17.9)		1 (1.8)	
C/T (*n* = 116)	46 (39.7)	> 0.99	15 (12.9)	> 0.99	4 (3.4)	> 0.99
T/T (*n* = 50)	21 (42.0)		7 (14.0)		2 (4.0)	

Abbreviations: ABCB1, ATP-binding cassette B1; aGVHD, acute graft-versus host disease; CYP3A4/5, cytochrome P450 isoforms 4 and 5.

Supplementary Figure

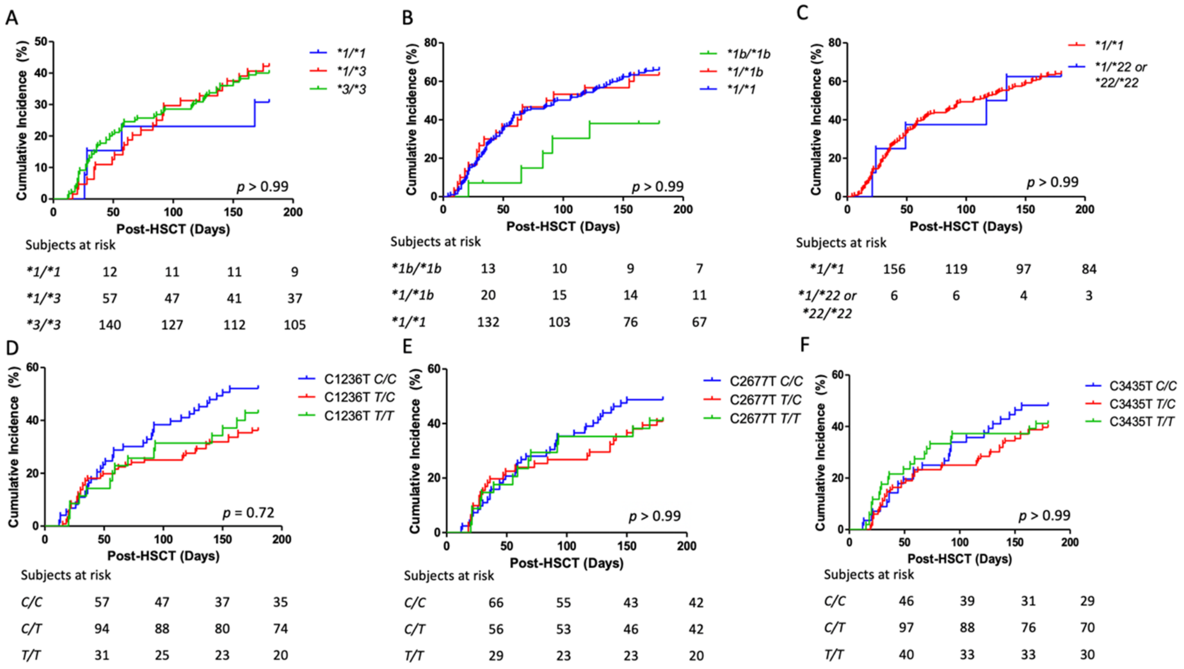

Figure S1. Cumulative incidence of aGVHD by *CYP3A4/5* and *ABCB1* genotypes. Cumulative incidence of all-grade aGVHD by each SNP is shown using Kaplan Meier curves where the y-axis denotes cumulative incidence of patients experiencing aGVHD, while the x-axis denotes days post-transplant. Subjects at risk on days +50, +100, +150, and +200 post-allogeneic HSCT are shown. *CYP3A5*3* (**A**), *CYP3A4*1b* (**B**), *CYP3A4*22* (**C**), *ABCB1* C1236T (**D**), *ABCB1* C2677T (**E**), and *ABCB1* C3435T (**F**) were evaluated for time to aGVHD occurrence. $p < 0.05$ was considered significant, and reported p values were adjusted for multiple comparisons using the Bonferroni method. Abbreviations: ABCB1, ATP-binding cassette B1; aGVHD, acute graft-versus host disease; CYP3A4/5, cytochrome P450 isoforms 4 and 5; HSCT, hematopoietic stem cell transplant.
